# Influence of Meso-Scale Pore Structure on Mechanical Behavior of Concrete under Uniaxial Compression Based on Parametric Modeling

**DOI:** 10.3390/ma15134594

**Published:** 2022-06-30

**Authors:** Hao Yang, Eryu Zhu, Lei Liu

**Affiliations:** School of Civil Engineering, Beijing Jiaotong University, Beijing 100044, China; yanghaobg@126.com (H.Y.); leiliu@bjtu.edu.cn (L.L.)

**Keywords:** meso-pore structure, concrete mesoscopic model, compressive elastic modulus, compressive strength

## Abstract

Existing concrete random aggregate modeling methods (CRAMMs) have deficiencies in in the parameterization of the mesoscale pore structure. A novel CRAMM is proposed, whose pore structure is determined by the pore gradation, total porosity, sub-porosity, and pore size of each pore gradation segment. To study the influence of pore structure on the mechanical properties of concrete, 25 mesoscopic concrete specimens with the same aggregate structure but different meso-scale pore structures are constructed and subjected to uniaxial compression tests. For the first time, the influence of sub-porosity of each pore gradation segment, average pore radius (APR), pore specific surface area (PSSA), and total porosity on concrete failure process, compressive strength, peak strain, and elastic modulus were quantitatively and qualitatively analyzed. Results indicate that the pore structure makes the germination and propagation of the damage in cement mortar show obvious locality and affects the formation and expansion of macroscopic cracks. However, it does not accelerate the propagation of the damage in cement mortar from the periphery to the center of the specimen, nor does it change the phenomenon that the ITZ is more damaged than other meso-components of concrete before peak stress. Macroscopic cracks occur in the descending section of the stress–strain curve, and the sudden drops in the descending section of the stress–strain curve are often accompanied by the generation and expansion of macroscopic cracks. The quadratic polynomial, exponential, and power functions can well fit the relationship between total porosity and compressive strength and the relationship between PSSA and compressive strength. The linear, exponential, and power functions can well reflect the relationship between total porosity and compressive modulus and the relationship between compressive modulus and PSSA. For concrete specimens with the same total porosity, the elastic modulus and strength show randomness with the increase in the sub-porosity of macropores and are basically not affected by the APR. Based on the grey relational analysis, the effects of pore structure parameters on the elastic modulus and compressive strength are in the same order: total porosity > T [k_1_,k_2_] > T [k_2_,k_3_] > T [k_3_,k_4_] > T [k_4_,k_5_] > AVR > PSSA. The order of influence of the pore structure parameters on the peak strain is: T [k_2_,k_3_] > T [k_1_,k_2_] > T [k_3_,k_4_] > T [k_4_,k_5_] > APR > PSSA > total porosity.

## 1. Introduction

Based on the analytic hierarchy process (AHP), the research level of concrete structures is classified into three levels: micro-scale (10^−6^~10^−3^ mm), meso-scale (10^−3^~100 mm), and macro-scale (3~4 times the maximum aggregate size) [1]. Concrete is a typical porous material with pores spanning the above three levels [2]. Statistics [3] show that the sub-porosity of pores with radii between 26.5 nm and 53 nm is about 1% in ordinary concrete, and the total porosity is generally greater than 8%. Total porosity represents the ratio of the volume of all pores in concrete to the volume of the concrete. The sub-porosity of the pores in a certain pore size range represents the ratio of the volume of the pores in the pore size range to the volume of the concrete [4]. Pore size is an indication of the size of a pore. For spherical pores, the pore size is usually expressed in radius or diameter. At present, there are mainly three ways to explore the effects of meso-scale pore structure on the mechanical behavior of concrete. The first is the theoretical analysis method. In recent years, many researchers have studied the effective mechanical properties of porous materials from the perspective of micromechanics of composite materials, and a variety of prediction models (Kemeny [5], Park [6], Feng XQ [7], and Spatschek [8]) have been proposed to analyze and calculate the effective modulus and effective strength. Mackenzie [9] used the isolated pore theory to calculate the analytical expressions of the effective shear modulus and bulk modulus of low-porosity materials, which did not consider the interaction among pores. The methods considering the interaction among pores include the self-consistent method [10], Mori–Tanaka method [11], etc. Du et al. [2] used a three-phase sphere model and a hollow cylindrical rod model to derive the relationship between effective strength and porosity and the relationship between effective peak strain and porosity. According to material science and the fracture mechanics of concrete, Jin et al. [12] analyzed the influence of pores on the surface energy and modulus, modified Griffith’s fracture theory, and then set up a compound–body model of pore structure. The results of the theoretical research need to be verified by experiments. The second method is the experimental method. Gao et al. [4] and Du Plessis A [13] measured the sub-porosity of pores of different sizes by an optical microscope, and studied the effect of sub-porosity on compressive strength. Zhao Jing [14] and B. Kondraivendhan [15] used the mercury intrusion method to study the effect of pore distribution and pore size on the strength and durability of concrete. Liu Huawei [16] applied 3D-printing technology to the study of concrete pore structure. In experimental research, many scholars [17,18,19,20] have tried a variety of innovative experimental methods and research methods. The research based on the experimental method proves that the use of total porosity to characterize the influence of meso-scale pore structure on the mechanical behavior of concrete is unscientific, and the influence of pore size and sub-porosity of pores in different pore size ranges cannot be ignored. However, the experimental method has an obvious defect in exploring the influence of pore structure parameters on concrete mechanical behavior; that is, it is difficult to achieve accurate control variables for pore structure parameters. For example, the current specimen preparation technology often changes the aggregate structure when changing the pore structure, which leads to changes in the mechanical behavior of concrete not only caused by changes in pore structure but also by changes in aggregate structure. The aggregate structure has a great effect on the mechanical properties of concrete [21,22]. This is an insurmountable defect of using experimental methods to establish the quantitative relationship between pore structure and concrete mechanical properties. The third method is the numerical simulation method. There are two main methods for the establishment of mesoscopic digital concrete: image-based model construction methods and parametric model construction methods. The former establishes a model based on the concrete meso-structure images obtained by the detection technology, which can accurately reflect the structural information of each constituent phase, such as shape, size, and distribution [23,24,25,26,27,28,29,30]. However, when applied to quantitatively analyze the influence of pore structure on concrete mechanical behaviors, it has a serious defect, that is, like the experimental method, it is difficult to change the pore structure parameters on the premise of keeping the aggregate structure unchanged. Parametric modeling methods firstly parameterize the meso-structure information of concrete and then build a model based on these parameters. By changing the structural parameters, concrete models that satisfy the control variables of the pore structure parameters can be constructed, which provides a solution to the above problems. The concrete random aggregate modeling method (CRAMM) is a typical representative of the parametric modeling method and has been widely used. Most existing CRAMMs model concrete as a two-phase material containing only aggregate and cement mortar or a three-phase material containing aggregate, cement mortar, and an interfacial transition zone (ITZ) [31,32,33,34,35,36,37]. A few CRAMMs take into account pores and treat concrete as a four-phase material [38,39]. However, most of them treat the pore structures simply as a structure composed of some randomly distributed single-graded oval or circular pores, which is quite different from the internal pore structure of concrete. Wang et al. [38] and Chen et al. [40] modeled the meso-scale pore structure as some randomly distributed circular or oval pores with a diameter of 2~4 mm and studied the influence of total porosity on the mechanical behaviors of concrete. Li et al. [41] modeled the pore structure in concrete as many randomly distributed circular or oval pores of a single size. Scholars are trying to explore new methods that can reasonably simulate the complex pore structure of concrete while taking into account the pore structure parameters of interest. For example, Zhu et al. [42] established a new pore structure establishment method based on fractal theory and studied the influence of total porosity on the mechanical behavior of concrete. The CRAMM based on fractal theory constructs the pore structure by setting the pore fractal dimension, maximum pore size, total porosity, and minimum pore size [43], so it cannot be applied to study the effects of pore size and sub-porosity of each graded segment on the mechanical behavior of concrete. There is no report on the establishment of concrete pore structure based on parameters such as pore gradation, total porosity, sub-porosity, and pore size of each pore gradation segment. Pore gradation refers to the combination of pores of different sizes in concrete [44]. However, sub-porosity is an important factor affecting the mechanical behaviors of concrete [45]. To sum up, the effect of meso-scale pore structure parameters on the mechanical behaviors of concrete needs to be further studied, especially the influence of sub-porosity and PSSA under the premise of scientific simulation of pore structure.

This paper aims to systematically explore the influence of pore structure parameters such as total porosity, sub-porosity of different graded segments, PSSA, and APR on the cracking process, peak strain, modulus, and compressive strength of concrete based on scientifically simulated pore structure. First, a novel CRAMM is established, which considers pore gradation, sub-porosity, and pore size of different pore gradation segments to characterize the complex pore structure of concrete. Based on the control variable method (CVM) and the measured data of pore structure parameters, 25 concrete random aggregate models (CRAMs) with different pore structures are constructed. Second, to explicitly model crack initiation and propagation, zero-thickness cohesive interface elements (CIEs) are inserted within the ITZ and mortar. Finally, these 25 CRAMs are subjected to quasi-static uniaxial compression experiments, and the influence of total porosity, sub-porosity, PSSA, and APR on the concrete cracking process, compressive strength, modulus, and peak strain are comprehensively analyzed.

## 2. Generation of CRAMs

### 2.1. Modeling of Aggregates

Studies have shown that concrete designed by Fuller’s ideal gradation curve [46] is expected to have optimized structural strength and density [47]. Based on the Walraven formula [48] for the 2D models derived from the Fuller curve, the percent passing the d_0_ sieve is shown in Equation (1).

(1) $$\begin{array}{l} P(d<d_{0})=P_{k}(1.065d_{0}^{0.5}d_{\max}^{-0.5}-0.053d_{0}^{4}d_{\max}^{-4}-0.012d_{0}^{6}d_{\max}^{-6}\\ \;\;\;\;\;\;\;\;\;\;\;\;\;\;\;\;\;\;\;\;\;\;\;\;\;\;\;-0.0045d_{0}^{8}d_{\max}^{-8}-0.0025d_{0}^{10}d_{\max}^{-10}) \end{array}$$

where d is the aggregate size. P(d < d_0_) is the percent passing the d_0_ sieve. P_k_ is the volume percentage of aggregates, usually taken as 0.75. d_max_ is the maximum aggregate size.

To simplify the calculation, only the aggregates with a diameter greater than 2.36 mm are simulated by drawing on the previous research experience [38,49,50], and it is assumed that smaller particles are mixed into the mortar. The same aggregate size distribution as Sadjad [51] and Wu et al. [34] is adopted in this paper, as shown in Table 1.

### 2.2. Pore Size Distribution

Scholars have adopted various methods to test the pore structure of concrete. Qin et al. [52] used CT scanning technology to test concrete specimens and found that pores are randomly distributed in concrete, and the pore size is log-normal or extreme value distribution. Gao et al. [4] used the quantitative stereoscopic image analysis method to measure pore structure parameters such as pore spatial distribution, total porosity, pore size, and sub-porosity. Chao et al. [53] and Wei et al. [54] also tested the pore distribution inside the concrete. With the deepening of the research on the pore structure of concrete, scholars agree that the influence of pore gradation, pore size, and sub-porosity of different pore gradation segments on the mechanical behaviors of concrete should not be ignored [12,55]. To realistically simulate the meso-scale pore structure in concrete and realize the research on the sub-porosity, this paper introduces pore gradation, total porosity, sub-porosity, and pore size of different pore-gradated segments to construct the pore structure in concrete. To explore the influence of total porosity on the mechanical behaviors, specimens with total porosity of 0%, 1%, 2%, 3%, and 4% are constructed in this paper. Pores are classified into large harmful pores, medium harmful pores, small harmful pores, and harmless pores according to the weakening degree of pores on the mechanical properties of concrete by Wu [55]. Concrete strength is mainly affected by large pores (>200 μm), while small pores (<10 μm) mainly affect the durability and permeability [56,57]. Based on the actual measurement data in references [4,52,53,54] and Hui Gao’s research experience [4], the radius of the pores established in this paper ranges from 0.15 mm to 0.8 mm and is divided into four graded segments, namely [k_1_,k_2_] = [0.15,0.25], [k_2_,k_3_] = [0.25,0.4], [k_3_,k_4_] = [0.4,0.6], [k_4_,k_5_] = [0.6,0.8]. It is assumed that the smaller pores are mixed with the mortar to form a homogeneous mortar. To study the effect of sub-porosity, the Lar-type specimens with relatively high macropore content, the Sma-type specimens with relatively high content of small pores, and the Mid-type specimen with relatively medium content of large and small pores were established for each total porosity, as shown in Table 2. To ensure the scientificity of the sub-porosity values, the sub-porosity values set in this paper are all within the range of sub-porosity measured in reference [54]. According to the CVM, specimens with different pore space distributions but the same total porosity, aggregate structure, pore size, and sub-porosity of each pore-gradated segments are constructed to explore the effect of pore space distribution on the mechanical behaviors of concrete. The relevant parameters of the concrete specimens established in this paper are shown in Table 2. The Specimen 0–1 that does not contain pores with a radius greater than 0.15 mm is considered as the reference specimen in this paper. The first digit of the sample name in Table 2 represents the total porosity.

### 2.3. Generation of Numerical Concrete Samples

**Figure 1:**
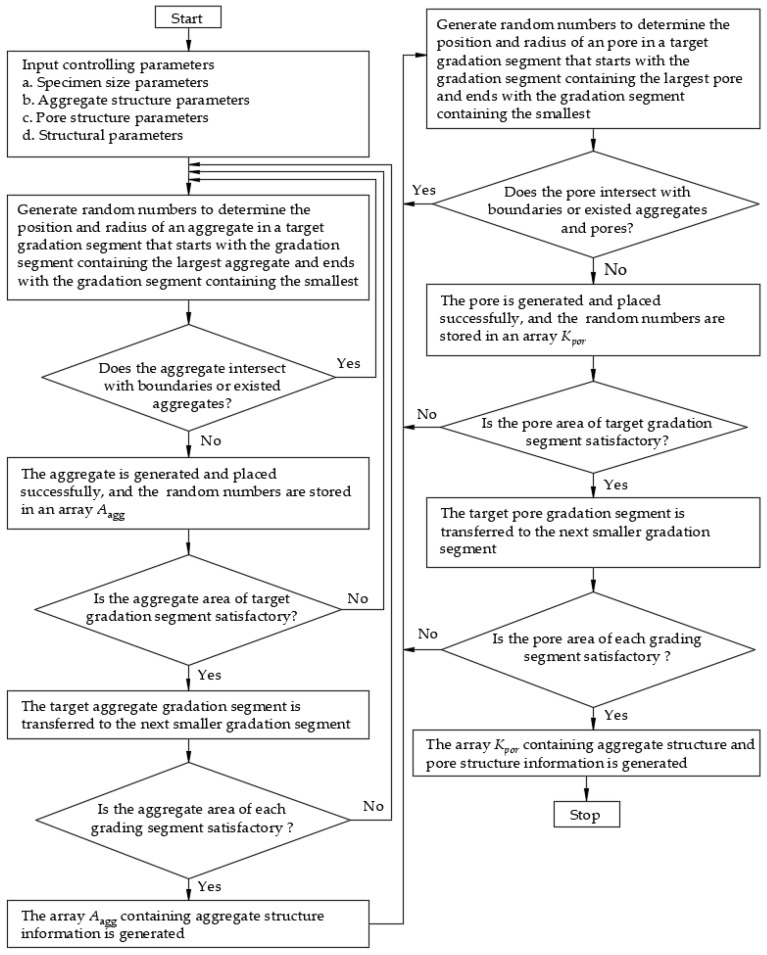
Flowchart of the aggregate and pore structure generation algorithm.

A new aggregate and pore generation algorithm is proposed, which introduces pore gradation, total porosity, pore size, and sub-porosity of each pore-gradated segments to characterize the pore structure. The flow chart is shown in Figure 1, in which the aggregate structure parameters include the aggregate area/volume ratio, aggregate type, aggregate shape, and the Fuller curve, etc. Pore structure parameters include shape, pore gradation, pore size, total porosity, and sub-porosity, etc., as described in Section 2.2.

Structural parameters include:

(1) The minimum distance δ_1_ between sample boundary and pore boundary.
(2) Minimum thickness of cement mortar layer δ_2_.
(3) The array *A*_agg_ has n rows and m columns; where n is the number of aggregates, m is equal to the number of random numbers of aggregate information. For two-dimensional circular aggregates, the random numbers for determining aggregate information include the x coordinate of the aggregate center, the y coordinate of the aggregate center, and the radius of the aggregate, so m = 3.
(4) The array *K_por_* has (n + k) rows and m columns, where k is equal to the number of pores. The first n rows of the array *K_por_* are equal to the array *A*_agg_.

The Boolean operations—“overlapping” and “subtraction”—are applied to generate mortar. Keeping the aggregate structure parameters unchanged and changing the pore structure parameters, concrete specimens with different pore structures and the same aggregate structure can be generated.

**Figure 2:**
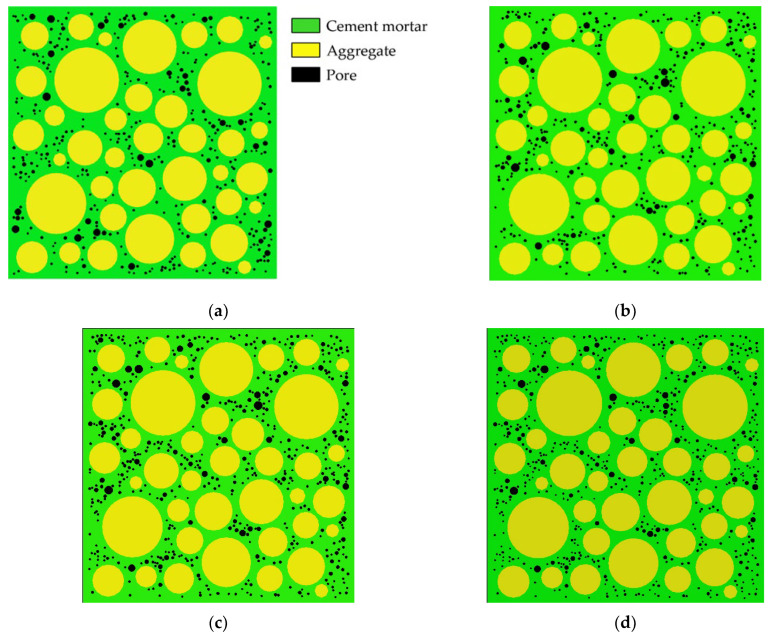
CRAMs with different sub-porosity but the same total porosity. (**a**) L-3-1 (total porosity = 3%); (**b**) M-3-1 (total porosity = 3%); (**c**) L-4-1 (total porosity = 4%); (**d**) 4-1-M (total porosity = 4%).

**Figure 3:**
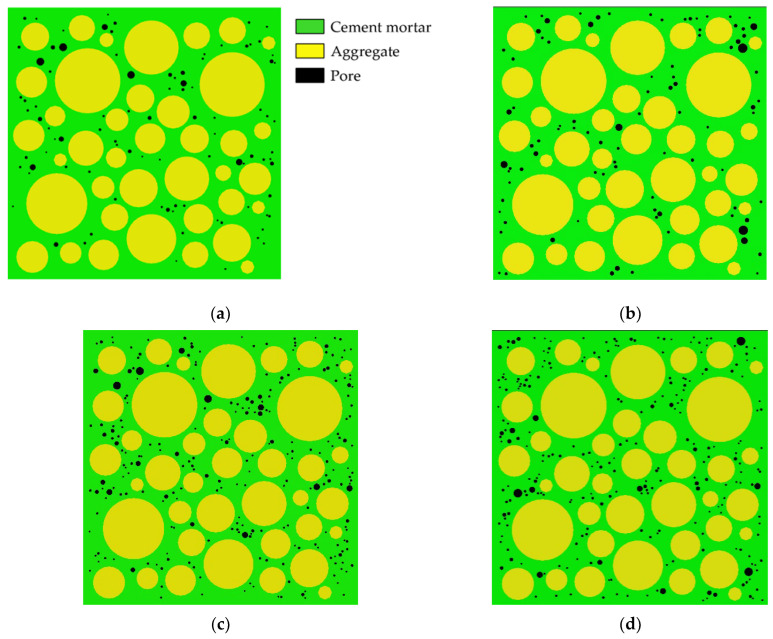
CRAMs with different spatial distributions but the same total porosity, and sub-porosity. (**a**) M-1-1 (total porosity = 1%); (**b**) M-1-2 (total porosity = 1%); (**c**) M-2-1 (total porosity = 2%); (**d**) M-2-2 (total porosity = 2%).

Aggregates are typically modeled as circles, ovals, or polygons. The mechanical properties of concrete are affected by aggregate shape, but not significantly [38]. Zhu Liang’s test [42] shows that the pores in concrete are mostly spherical. Therefore, both pores and aggregates are modeled as circular in this paper. Interactions between pores are ignored. Using the aggregate structure parameters and pore structure parameters described in Section 2.1 and Section 2.2, where the aggregate area ratio is 45% and δ_1_ = δ_2_ = 0.5 mm, 25 CRAMs with different pore structures are constructed based on the CVM. The size of all specimens established is 50 mm × 50 mm, and Table 2 shows the pore structure parameters, in which APR and PSSA are also important pore structure parameters [58,59,60]. Figure 2 shows some CRAMs with different sub-porosity but the same total porosity and aggregate structure where the yellow circles represent aggregate, the green area represents the mortar, and black circles represent pores. Figure 3 shows some CRAMs with different spatial distributions but the same total porosity, sub-porosity, and aggregate structure.

ITZ, which has an important influence on the development of micro-cracks in concrete [22,61], has always been a difficult point in simulation due to its small thickness (10~50 μm) [62]. For the ITZ simulation of mesoscopic concrete, some scholars [29,63] did not consider it, and some scholars determined it by projection in the background grid [64] or lattice model [65], but the obtained ITZ scale is in the order of millimeters, which is quite different from the real ITZ. More scholars [35,38,50,66] have used zero-thickness cohesive elements to simulate ITZ, which is more realistic and enables the cracks to be expressed explicitly. 

The bilinear traction–separation law is adopted for zero-thickness CIEs:

(2) $$\begin{array}{cc} t_{n}=\left\{\begin{array}{cc} \frac{t_{n}^{0}}{\delta_{n}^{0}}\delta_{n} & \left(\delta_{n}\leq \delta_{n}^{0}\right)\\ t_{n}^{0}\frac{\delta_{n}^{f}-\delta_{n}}{\delta_{n}^{f}-\delta_{n}^{0}} & \left(\delta_{n}>\delta_{n}^{0}\right) \end{array}\right. & t_{t}=\left\{\begin{array}{cc} \frac{t_{t}^{0}}{\delta_{t}^{0}}\delta_{t} & \left(\delta_{t}\leq \delta_{t}^{0}\right)\\ t_{t}^{0}\frac{\delta_{t}^{f}-\delta_{t}}{\delta_{t}^{f}-\delta_{t}^{0}} & \left(\delta_{t}>\delta_{t}^{0}\right) \end{array}\right. \end{array}$$

where *t_n_* and *t_t_* are the normal traction and tangential traction on adjacent virtual surfaces, respectively. $t_{n}^{0}$ and $t_{t}^{0}$ are the maximum traction values in the normal and tangential directions, respectively. $\delta_{n}$ and $\delta_{t}$ are normal and shear separations, respectively. $\delta_{n}^{0}$ and $\delta_{t}^{0}$ are the crack interface opening displacement values corresponding to the maximum stress in the normal and tangential directions, respectively. $\delta_{n}^{f}$ and $\delta_{t}^{f}$ are normal and tangential displacement jump at completion of debonding, respectively.

The CIEs can be damaged and destroyed by excessive tension, shearing, or a combination. The quadratic nominal stress law is used to judge damage initiation in this paper. As the damaged state of the CIEs progresses, the material begins to soften. The scalar damage variable *D* is defined to describe the damage evolution process, which monotonically varies from 0 to 1:

(3) $$D=\left\{\begin{array}{c} \begin{array}{cc} \delta_{m}^{f}(\delta_{m}^{\max}-\delta_{m}^{0}), & \delta_{m}^{\max}\geq \delta_{m}^{0} \end{array}\\ \begin{array}{cc} 0, & \delta_{m}^{\max}\leq \delta_{m}^{0} \end{array} \end{array}\right.$$

where $\delta_{m}^{0}$, $\delta_{m}^{f}$ and $\delta_{m}^{\max}$ are the effective separations at the damage initiation, the effective separations at the complete failure, and the maximum effective separation obtained during the loading history.

The traction for the damage state is a function of the traction without damage and the damage variable:

(4) $$\begin{array}{l} t_{n}=\left\{\begin{array}{l} \begin{array}{cc} (1-D)k_{n}\delta_{n} & \left(tension\right) \end{array}\\ \begin{array}{cc} k_{n}\delta_{n} & \left(compression\right) \end{array} \end{array}\right.\\ t_{t}=(1-D)k_{t}\delta_{t} \end{array}$$

where $k_{n}$ and $k_{t}$ are the initial stiffness in the normal direction and the tangential direction.

**Figure 4:**
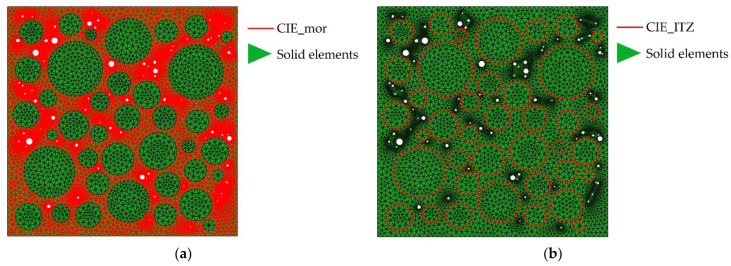
The distribution of CIEs. (**a**) CIE_mor; (**b**) CIE_ITZ. The white circles represent pores.

The pre-processing functionality in ANSYS is used to mesh the aggregate and cement mortar and the triangular elements are applied to make the cracking path more real. For the ordinary concrete as the research object of this paper, the aggregates are generally not destroyed when the concrete is damaged. Therefore, the four-node zero-thickness cohesive interface elements (CIEs) are only inserted in the cement mortar (called CIE_mor) and the ITZ (called CIE_ITZ) by the CIEs insertion scheme, as shown in Figure 4. The CIEs insertion program is completed collaboratively using ABAQUS and python on the basis of the relevant literature [61,67] and existing algorithms [68].

### 2.4. Numerical Validation

**Figure 5:**
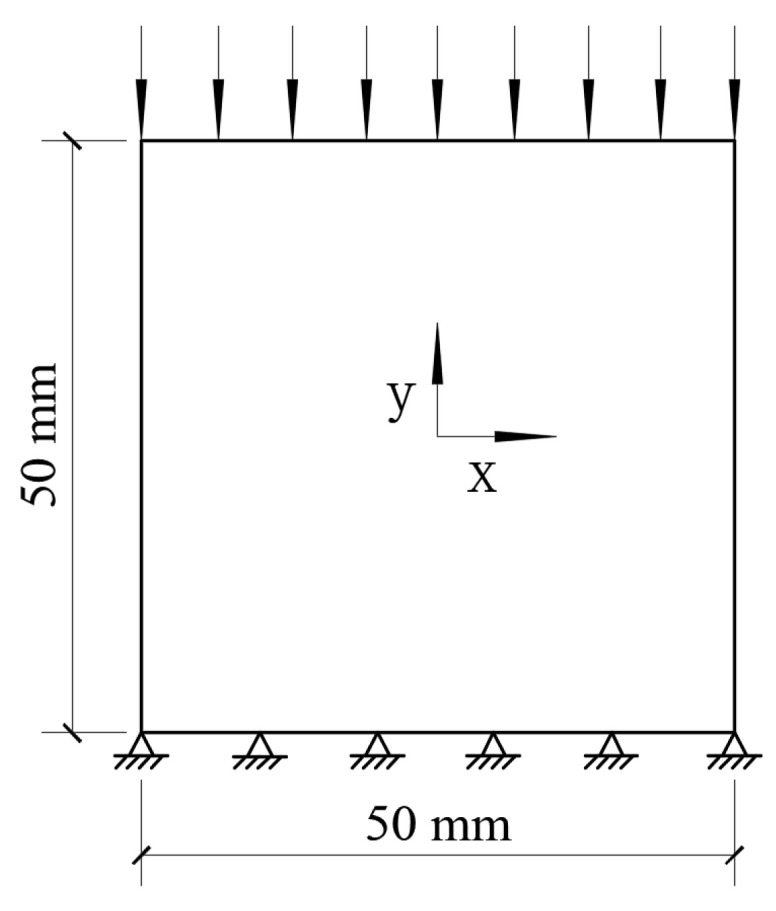
Loads and boundary conditions for quasi-static uniaxial compression experiments.

**Figure 6:**
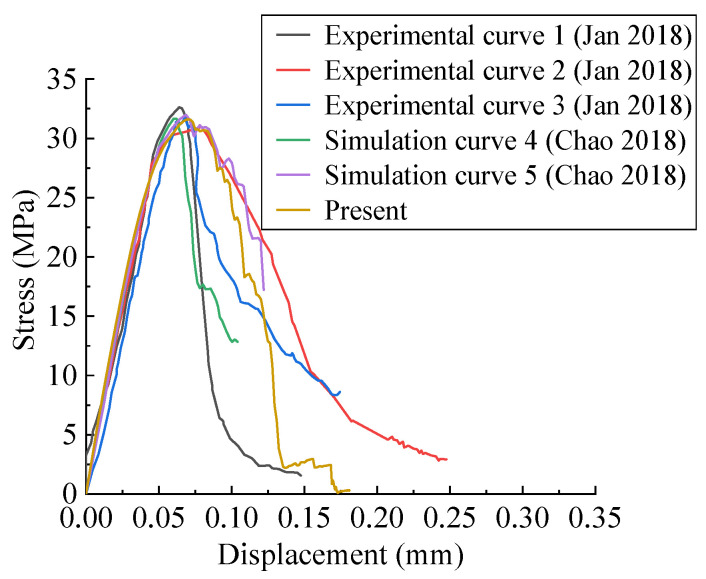
Comparison of stress–strain curves of uniaxial compression experiments.

To verify the effectiveness of the CRAMs established in this paper in characterizing the mechanical behaviors of concrete, based on the real specimens prepared by Sucor-zewski [69], a 50 mm × 50 mm numerical specimen was established in this paper. Wu et al. [34] also established some 50 mm × 50 mm numerical specimens based on Sucorzewski’s specimens. The aggregate size distribution is shown in Table 1. A quasi-static uniaxial compression experiment was carried out on it. The boundary conditions of the uniaxial compression experiment are that the lower boundary is fixed in the X and Y directions. A compressive load with displacement control is applied on the upper boundary, as shown in Figure 5. The material properties used in this paper are determined by referring to the relevant literature [34,35,38,70] and extensive trial calculations, as shown in Table 3. The calculated results are compared with the experimental results obtained by Suchorzewski [69] and the numerical simulation experimental results obtained by Wu et al. [34], as shown in Figure 6. The experimental stress–strain curves of concrete specimens [69] prepared with the same material, aggregate gradation, and preparation conditions indicate a big scatter in the descending section. Three representative stress–strain curves are selected to shown in Figure 6, namely curve 1 with obvious brittleness, curve 2 with obvious quasi-brittleness, and curve 3 with moderate quasi-brittleness. All experimental curves in Ref. [69] are located between curve 1 and curve 2, such as curve 3. Simulation curve 4 and simulation curve 5 in Figure 6 are the stress–strain curves of two CRAMs with different aggregate space distribution but the same aggregate gradation and material properties [34]. It can be seen from Figure 6 that the descending section of the simulation curve 4 shows strong brittleness, which is close to the experimental curve 1, and the descending section of the simulation curve 5 shows a strong quasi-brittleness, which is close to the experimental curve 2. As shown in Figure 6, the stress–displacement curve obtained in this paper has a good similarity with the experimental curves [69] and simulated curves [34] in the ascending, softening, and peak segments, and the descending section is located between curve 1 and curve 2. The elastic modulus and strength of these specimens are shown in Table 4, in which the average value is taken as the calculated value. It can be seen that the simulation results in this paper are better than the results of Wu et al. [34]; that is, the strength is at the same error level, and the error of the elastic modulus is smaller.

As shown in Figure 6, the descending sections of these stress–strain curves exhibit a large dispersion. The descending section is very sensitive to the aggregate structure [22] and material properties [35], and it is found that in this paper the pore structure also has a great influence on it. This may be the reason for the large dispersion of the descending section. The comparison results indicate that the CRAMs constructed in this paper can well simulate the mechanical responses of concrete under uniaxial compressive load with the calibrated material parameters as listed in Table 3.

## 3. Numerical Experiments and Analysis of Results

Quasi-state uniaxial compression experiments are performed on 25 mesoscopic specimens with different pore structures, as shown in Table 2.

### 3.1. Analysis of Cracking Process

Concrete cracking is a process in which microscopic cracks are initiated, expanded, connected, and finally form macroscopic cracks [71]. Based on previous research experience [72,73] and the numerical experiment results obtained in this paper, the cracking process is classified into three levels: the initiation stage of micro-cracks, the stable development stage of micro-cracks, and the unstable crack propagation stage. Cracking at each stage shows different characteristics and is significantly affected by the pore structure. According to the visual cracking process obtained from the experiments in this paper, the influence of the pore structure on the concrete failure process is analyzed. 

#### 3.1.1. Failure Process of the Concrete Specimen with Porosity of 0%

**Figure 7:**
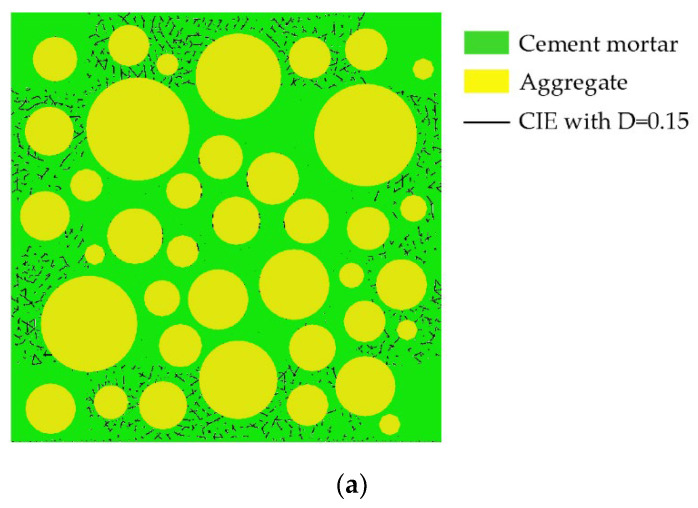

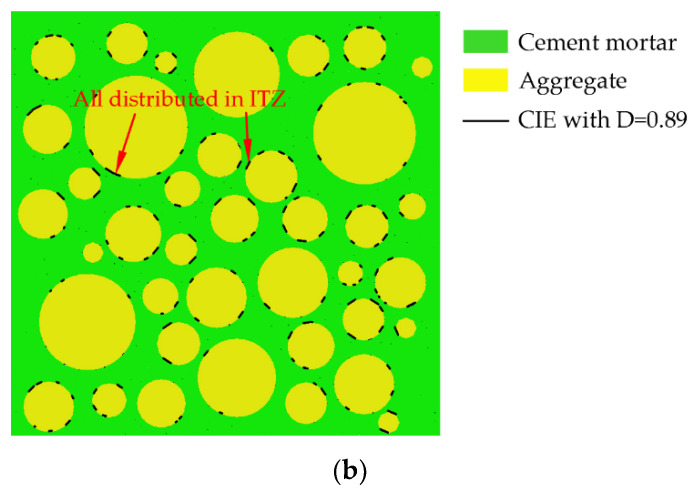
Distribution of CIEs for D = 0.15 and D = 0.89 when σ/σ_max_ ≈ 0.3. (**a**) D = 0.15; (**b**) D = 0.89. The junction of the yellow area representing aggregate and the green area representing cement mortar is ITZ.

**Figure 8:**
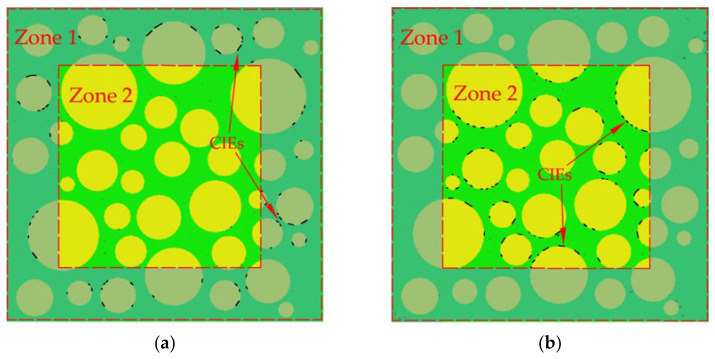
Distribution of CIEs for D = 0.55 and D = 0.69 when σ/σ_max_ ≈ 0.136. (**a**) D = 0.55; (**b**) D = 0.69.

1. The initiation stage of micro-cracks (σ/σ_max_ < 0.3~0.5);

The damage degree of the CIE_ITZ and CIE_mor characterized by the damage variable D characterizes the damage degree of the ITZ and cement mortar to a certain extent. From the visual cracking process, it can be seen that the micro-cracks at this stage first initiate at the periphery of the specimen and then expand to the center. The damage propagation of the ITZ from the periphery to the center of the specimen is much faster than that of cement mortar. The reason may be that the stress concentration at ITZ is serious, and the strength of the ITZ is about 33~67% of mortar [74]. Most of the damage variables of CIE_mor in the later stage of this stage are no more than 0.55, which are distributed on the periphery of the specimen. The distribution of CIEs with D = 0.15 when σ/σ_max_ ≈ 0.3 are shown in Figure 7a where the black line segments represent CIEs. It can be seen that almost all CIEs with D = 0.15 are distributed in the mortar around the specimen. This indicates that the damage of the cement mortar during this stage has not extended to the center of the specimen. Most damage variables for CIE_ITZ are centered between 0.4 and 0.96. Figure 7b shows the distribution of the CIEs with D = 0.89 when σ/σ_max_ ≈ 0.3, from which it can be seen that all the CIEs with D = 0.89 are distributed in the ITZ. This indicates that the damage of the ITZ at this stage has extended to the center of the specimen. The damage of the ITZ in Figure 7b has expanded to the center of the specimen and does not show the expansion process. Figure 8 shows the CIEs with D = 0.69 and D = 0.55 when σ/σ_max_ ≈ 0.136, from which it can be seen that CIEs with D = 0.69 are mainly distributed in zone 1, and CIEs with D = 0.55 are mainly distributed in zone 2. Zone 1 represents the peripheral area of the specimen, and zone 2 represents the central area of the specimen. In the stage of damage development, the damage of the first damaged area will continue to increase with the increase in the load. Therefore, the damage degree of the first damaged area will be greater than that of the later damaged area. From this, it can be judged that the damage of the ITZ extends from zone 1 to zone 2, which shows the damage process extending from the periphery to the center of the specimen.

**Figure 9:**
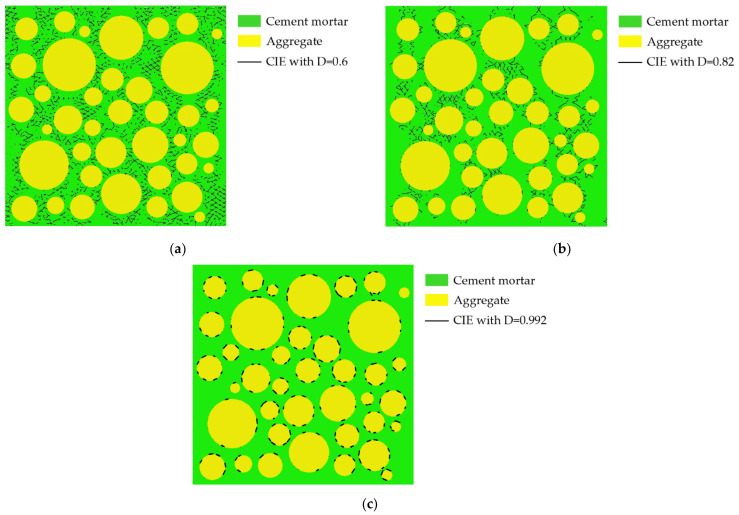
Distribution of CIEs for D = 0.6, D = 0.82, and D = 0.992 when σ/σ_max_ ≈ 0.81. (**a**) D = 0.6; (**b**) D = 0.82; (**c**) D = 0.992.

2. The stable development stage of micro-cracks (0.3~0.5 < σ/σ_max_ < 0.75~0.9);

The internal cracks of the ITZ and cement mortar continue to develop as the load increases. Figure 9a shows the distribution of the CIEs with D = 0.6 when σ/σ_max_ ≈ 0.81. Comparing Figure 7a and Figure 9a, it can be found that the damage expansion process from the periphery to the center of the specimen has been completed during this stage. Most of the damage variables of CIE_mor are less than 0.987. The CIEs with D = 0.82 when σ/σ_max_ ≈ 0.81 are shown in Figure 9b. Most damage variables for CIE_ITZ are centered between 0.95 and 0.998. The CIEs with D = 0.992 are shown in Figure 9c. It can be seen that in the later stage of this stage, the damage level of the mortar is still smaller than that of the ITZ.

**Figure 10:**
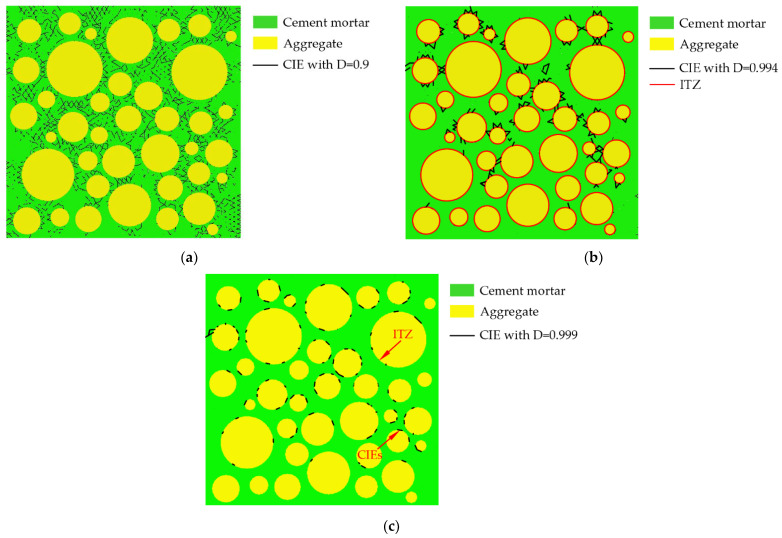
Distribution of CIEs for D = 0.9, D = 0.994 and D = 0.999 when σ/σ_max_ ≈ 0.999 (ε < ε_c_). (**a**) D = 0.9; (**b**) D = 0.994; (**c**) D = 0.999.

3. The unstable crack propagation stage (σ/σ_max_ > 0.75~0.9);

The CIEs with D = 0.9 when σ/σ_max_ ≈ 0.999 (ε < peak strain ε_c_) are shown in Figure 10a. Comparing Figure 9b and Figure 10a, it can be seen that the cracks in the mortar greatly increase under high stress. When approaching the ultimate stress, the maximum damage variable of CIE_mor reaches about 0.994. The CIE_mor with D = 0.994 when σ/σ_max_ ≈ 0.999 (ε < ε_c_) are shown in Figure 10b, where the black line segment represents the CIE, and the red line segment represents the ITZ. It can be seen that almost all the heavily damaged areas in the mortar are concentrated near the ITZ. Many scholars [72,75] speculate that the severely damaged areas in the cement mortar are caused by cracks in the ITZ propagating into the mortar. Most damage variables for CIE_ITZ are centered between 0.995 and 0.9995, and the maximum has reached about 0.99965. The CIEs with D = 0.999 when σ/σ_max_ ≈ 0.999 (ε < ε_c_) are shown in Figure 10c. It can be seen that almost all CIEs with D = 0.999 are distributed in the ITZ. From this, it can be inferred that the damage degree of the mortar is still smaller than that of the ITZ until the ultimate stress is reached.

**Figure 11:**
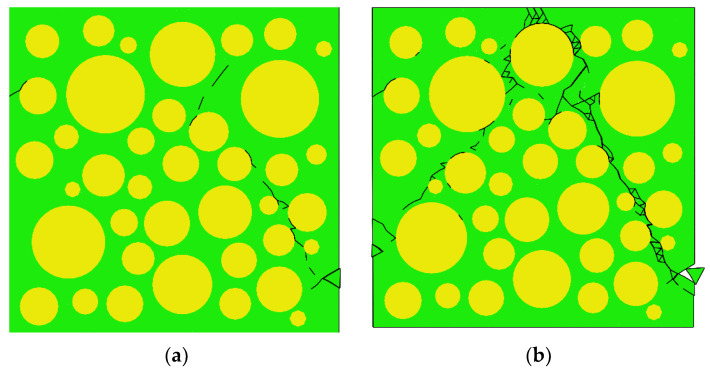
Distribution of CIEs for D = 1 when σ/σ_max_ ≈ 0.34 (ε > ε_c_) and the macroscopic cracks when the specimen finally fails. (**a**) D = 1; (**b**) The final crack paths.

After reaching the ultimate stress, macroscopic cracks form first in the most severely damaged area of the ITZ, and quickly penetrate with the surrounding cracks in the ITZ and cement mortar, as shown in Figure 11a. With the generation of macroscopic cracks, the stress drops rapidly. The final failure of the specimen is shown in Figure 11b, with the formation of two intersecting macroscopic cracks.

#### 3.1.2. Cracking Process of the CRAMs with Pores

Taking specimen L-2-1 as an example, the failure process analysis of the concrete specimen with pores is illustrated.

**Figure 12:**
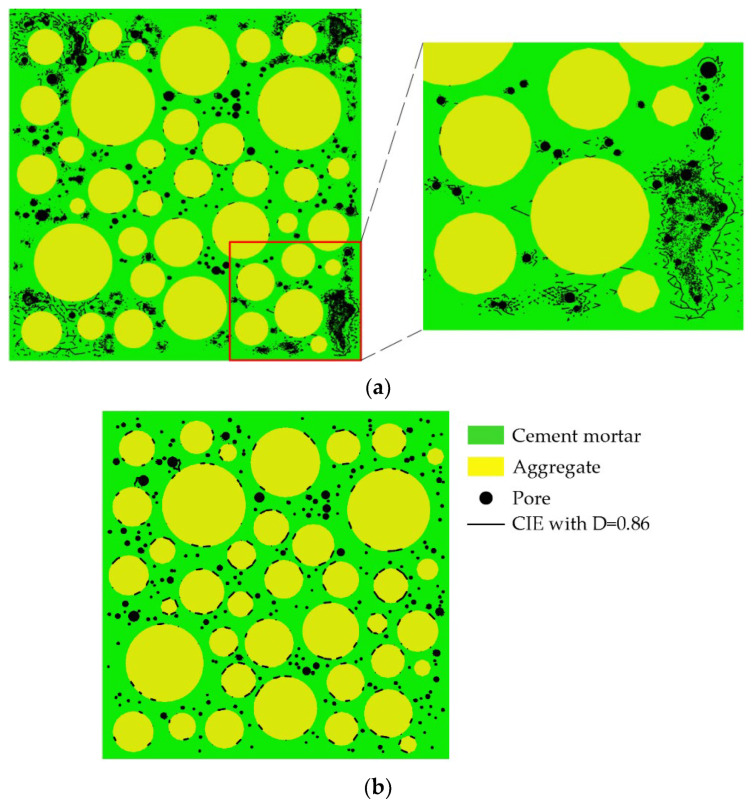
Distribution of CIEs for D = 0.27 and D = 0.86 when σ/σ_max_ ≈ 0.36. (**a**) D = 0.27; (**b**) D = 0.86.

1. The initiation stage of micro-cracks (σ/σ_max_ < 0.3~0.5);

The CIEs with D = 0.27 and D = 0.86 when σ/σ_max_ ≈ 0.36 are shown in Figure 12a,b, where the black circles represent pores, and the black line segments represent CIEs. As shown in Figure 12a, for the concrete specimens with pores, the damage in the cement mortar first occurs in the pore-concentrated area at the periphery of the specimen, which is caused by the stress concentration in the pore-concentrated area. At this stage, the failure process of the concrete specimens with pores is basically the same as that of the concrete specimen without pores. The damage also shows an obvious expansion law from the periphery to the center of the sample. Later in this stage, the damage of the ITZ has expanded to the center of the specimen, as shown in Figure 12b, while the damage of the mortar is still distributed on the periphery of the specimen, as shown in Figure 12a. From this, the presence of pores does not accelerate the propagation of the damage in the mortar from the periphery to the center of the specimen.

2. The stable development stage of micro-cracks (0.3~0.5 < σ/σ_max_ < 0.75~0.9)

**Figure 13:**
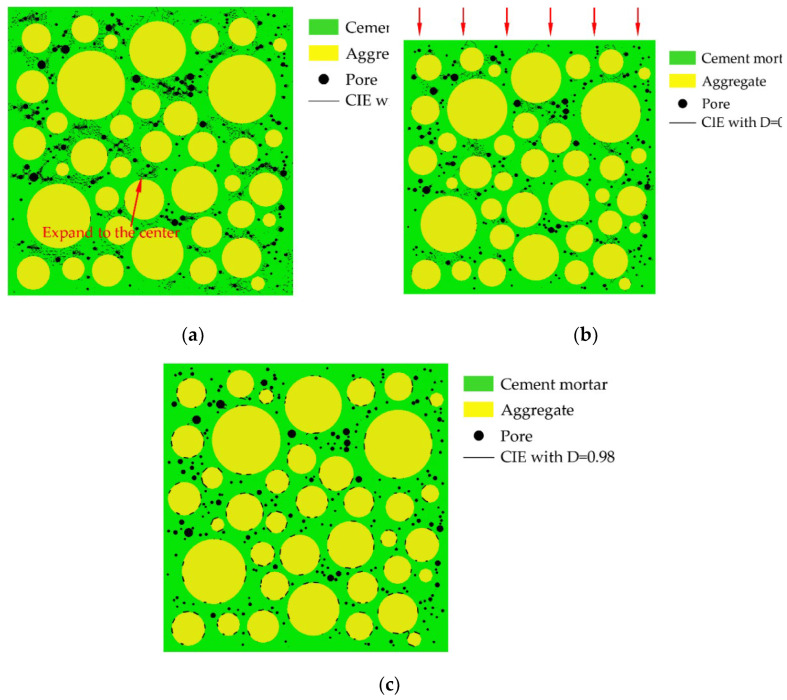
Distribution of CIEs for D = 0.6, D = 0.8, and D = 0.98 when σ/σ_max_ ≈ 0.84. (**a**) D = 0.6; (**b**) D = 0.8; (**c**) D = 0.98.

The CIEs with D = 0.6 when σ/σ_max_ ≈ 0.84 are shown in Figure 13a. Comparing Figure 12a and Figure 13a, it can be found that, like the specimen without pores, the damage in the cement mortar also spreads to the center of the specimen at this stage. Comparing Figure 9a and Figure 13a, it can be found that the pore structure makes the damage in the mortar more localized. This may be due to the damage and deformation of the cement mortar around the pores, releasing the stress of the cement mortar at other locations. The damage variables of CIE_mor are almost no more than 0.94. Figure 13b shows the CIEs with D = 0.8 when σ/σ_max_ ≈ 0.84, where the red arrow represents the load direction. The severely damaged areas in the mortar are mainly concentrated on the bearing paths parallel to the load direction, as shown in Figure 13b. The damage variables of CIE_ITZ are mainly distributed in the range of 0.84~0.996. The CIEs with D = 0.98 when σ/σ_max_ ≈ 0.84 are shown in Figure 13c. It can be seen that even if there are pores, the most severely damaged area in the concrete specimen remains the ITZ.

3. The unstable crack propagation stage (σ/σmax > 0.75~0.9).

**Figure 14:**
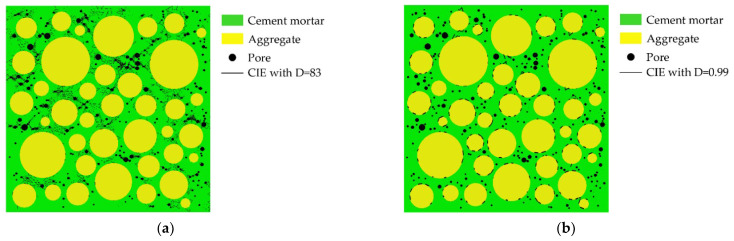
Distribution of CIEs for D = 0.83 and D = 0.99 when σ/σ_max_ ≈ 0.989, ε > ε_c_. (**a**) D = 83; (**b**) D = 0.99.

Damage in ITZ and mortar develops rapidly under high stress. The CIEs for D = 0.83 when σ/σ_max_ ≈ 0.989(ε < ε_c_) are shown in Figure 14a. Comparing Figure 10a and Figure 14a, it can be seen that the damage of the cement mortar of the specimen with pores still shows strong localization at this stage. The CIEs with D = 0.99 when σ/σ_max_ ≈ 0.989(ε < ε_c_) are shown in Figure 14b. It can be seen from Figure 14b that the damage of the mortar is still smaller than that of the ITZ before reaching the ultimate stress.

**Figure 15:**
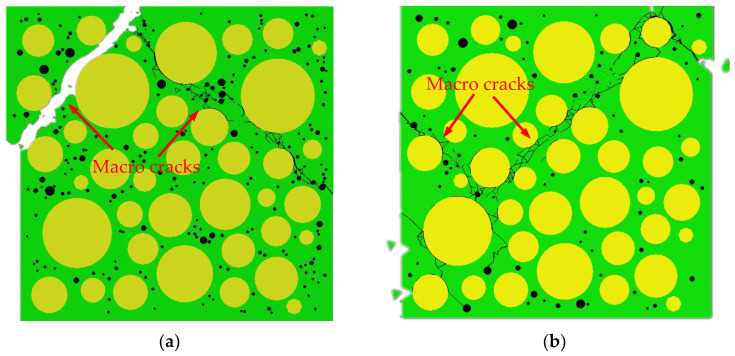

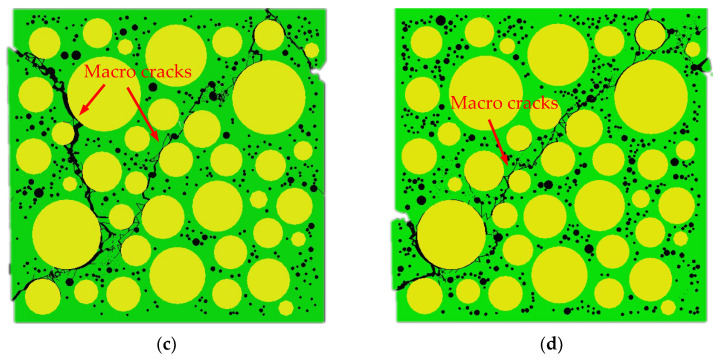
Cracking of some specimens with final failure. (**a**) L-2-1; (**b**) L-1-2; (**c**) L-3-2; (**d**) 4-2-L.

**Figure 16:**
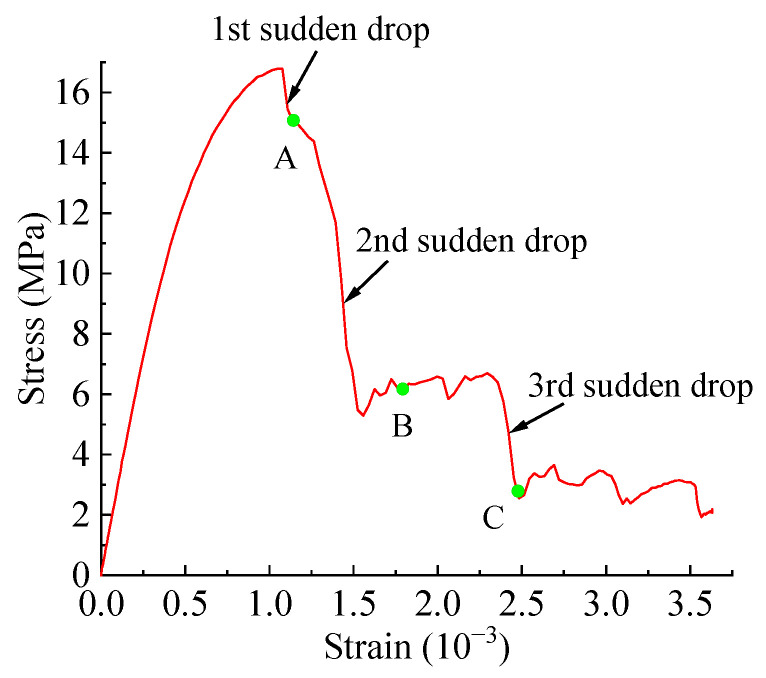
Stress–strain curve of sample S-4-1.

**Figure 17:**
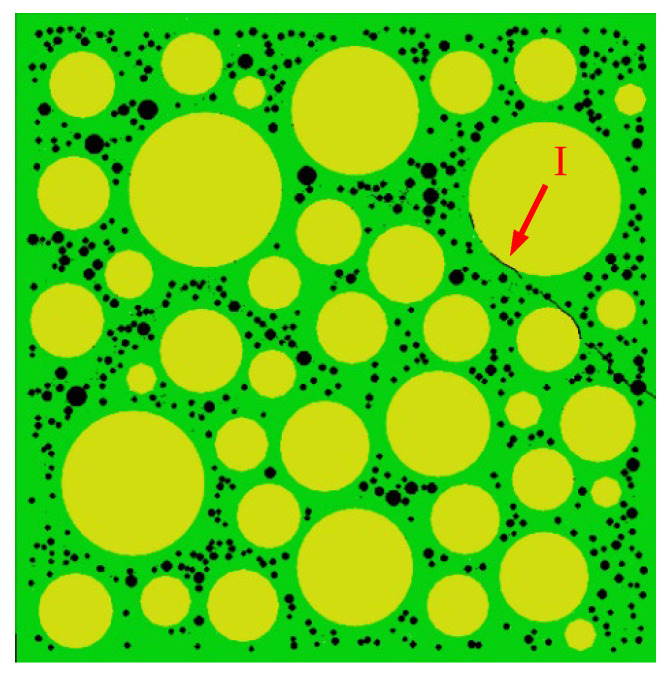
Cracks of specimen S-4-1 at point A.

**Figure 18:**
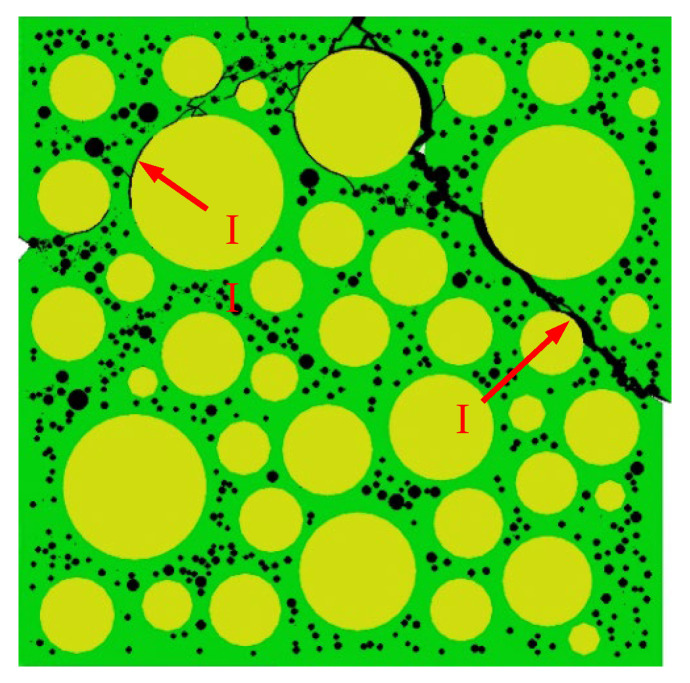
Cracks of specimen S-4-1 at point B.

**Figure 19:**
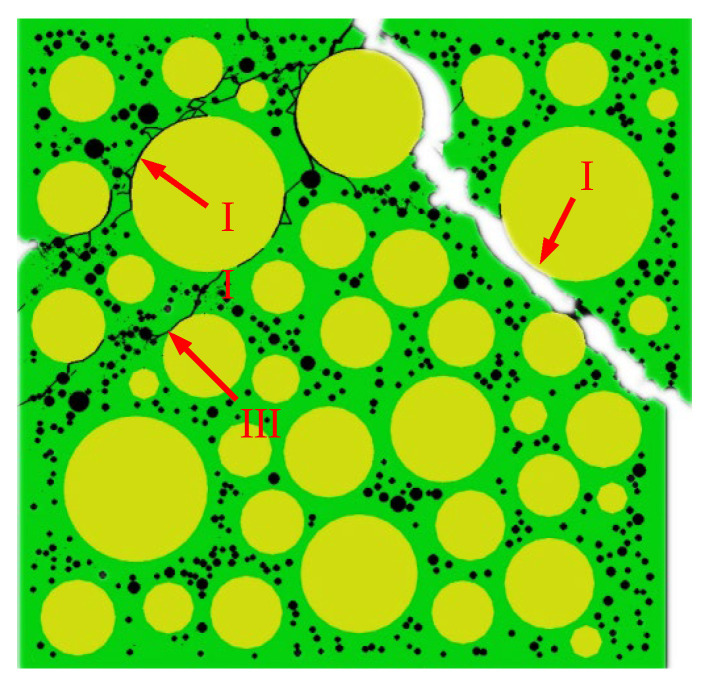
Cracks of specimen S-4-1 at point C.

Figure 15 shows the final failure of some concrete specimens with different pore structures but the same aggregate structure, from which it can be found that the macroscopic cracks are heavily influenced by the pore structure. Combining the process of generation and development of macroscopic cracks with the stress–strain curve of the specimen, it is found that the sudden drops in the descending section of the stress–strain curve are often accompanied by the generation and expansion of macroscopic cracks. Taking specimen S-4-1 as an example, Figure 16 shows its stress–strain curve, from which it can be seen that there are three sudden drops in the descending section. Figure 17 shows the cracks of the specimen after the first sudden drop (corresponding to point A marked in Figure 16), from which it can be seen that the first sudden drop is caused by the formation of the first macroscopic crack marked I in Figure 17. Figure 18 shows the cracks of the specimen after the second sudden drop in stress (corresponding to point B marked in Figure 16), from which it can be seen that the second sudden drop is caused by the development of the first macroscopic crack and the generation of the second macroscopic crack marked II in Figure 18. Figure 19 shows the cracks of the specimen after the third sudden drop in stress (corresponding to point C marked in Figure 16), from which it can be seen that the third sudden drop is caused by the development of the second macroscopic crack and the generation of the third macroscopic crack marked III in Figure 19. This may be caused by the sudden reduction in the load-bearing area of the specimen due to the sudden generation and development of macroscopic cracks.

**Figure 20:**
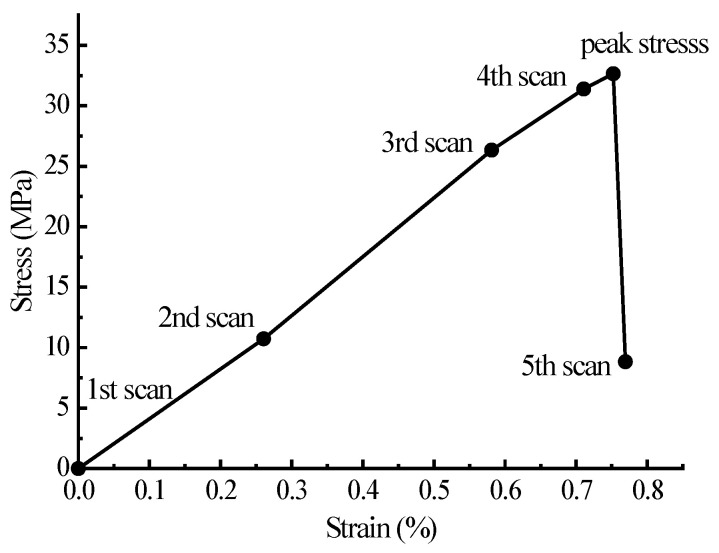
Scan sequence of 5 scans [76].

In recent years, more and more attention has been paid to the experimental research of concrete meso-structure. Using CT technology to observe and study internal cracks in concrete has gradually become a research hotspot. Dang Fanning et al. [76] used CT technology to observe the internal cracking process of concrete under uniaxial compressive load. The scan sequence is shown in Figure 20, and the obtained CT image can been seen in the reference [76]. It can be seen from the CT images that before the peak stress, no macroscopic cracks are formed inside the concrete, which is the same as the results in this paper. The 5th scan after the peak stress shows that the sharp drop in peak stress is accompanied by the formation of macroscopic cracks. However, due to insufficient stiffness of the loading equipment, it is difficult to measure the development process of macroscopic cracks in the descending section of the specimen. In the fifth scan, a number of macroscopic cracks have been generated, which is equivalent to the state of point C in Figure 16 of the simulated test in this paper. This not only proves the correctness of the conclusions of this paper, but also proves the superiority of this study.

**Figure 21:**
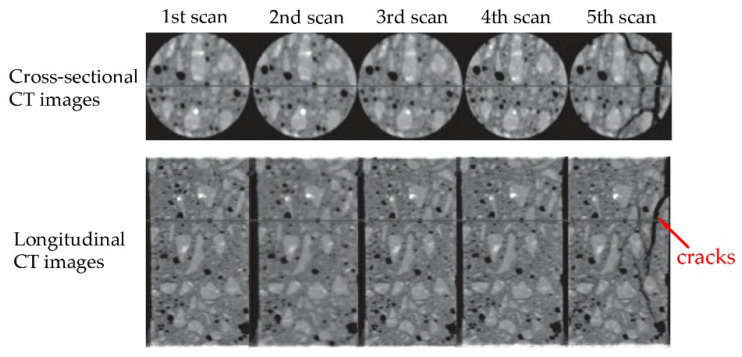
CT images [76].

The stress–strain curves of the 25 specimens established in this paper are shown in Figure 21. Combining Figure 21 and Table 5, it can be seen that the peak stress and modulus decrease with increasing total porosity, indicating that the total porosity has a great influence on the ascending segment of the stress–strain curve. However, the descending segments of the stress–strain curves show obvious randomness, even for specimens with the same total porosity. Visualizing the cracking process shows that after the occurrence of macroscopic cracks, the load is mainly borne by the concrete in the uncracked area and the cement mortar and aggregate in the cracked area through mutual friction and occlusion. Macroscopic cracks will occur soon after the stress–strain curve enters the descending section, and the generation and development of macroscopic cracks are extremely random, which may be the reason for the large difference and significant randomness of the stress–strain descending section.

### 3.2. Effect of Pore Structure on Compressive Strength of Concrete

The compressive strength, elastic modulus, and peak strain of the 25 specimens are shown in Table 5. The secant modulus at 50% of the peak stress in the ascending segment of the stress–strain curve is regarded as the elastic modulus [72,77], which is about the working stress of the concrete structure in the service stage [72]. The analysis, discussion, and conclusions based on the calculation results of this paper all imply a premise, that is, within the range of parameter values studied in this paper. For example, a conclusion drawn in this paper is that the quadratic polynomial function can well fit the relationship between the modulus and the total porosity. The implicit premise is that the applicable range of this conclusion is that the total porosity is in the range of 0~4%. It is hereby declared, and no special explanation will be made later.

**Figure 22:**
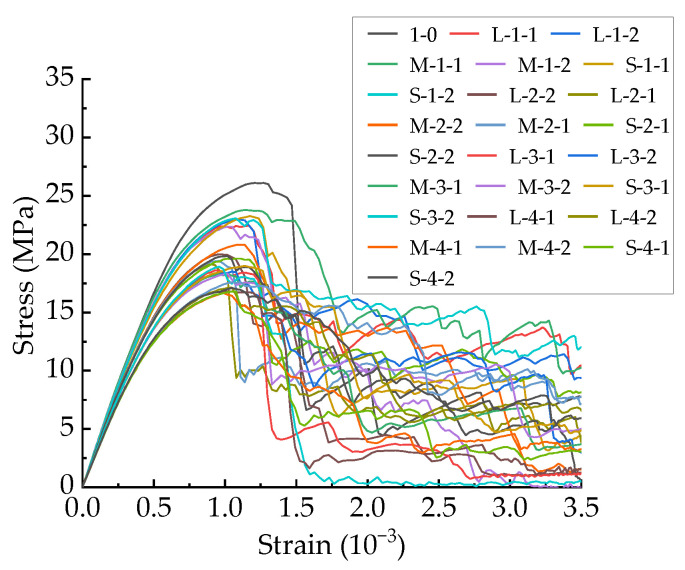
Stress–strain curves of 25 CRAMs.

**Figure 23:**
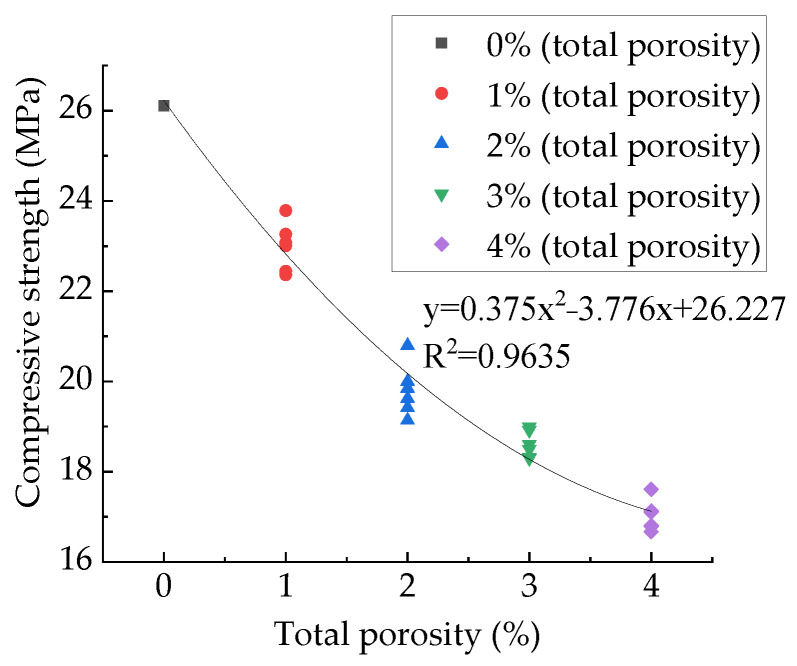
Relationship between compressive strength and total porosity.

In existing studies, the relationship between the pore structure and mechanical behaviors of concrete is usually described by polynomial relationships [4,57], exponential relationships [41,78], and power function relationships [2,79]. However, the previous studies have not achieved control variables for pore structure. Changes in concrete mechanical behaviors are caused by changes in pore structure and aggregate structure. To verify the rationality of these three functional relationships in describing the relationships between concrete mechanical behaviors and pore structure, this paper uses these three functions to fit the relationships between concrete mechanical parameters and pore structure parameters. Figure 22 shows the relationship between compressive strength and total porosity. Table 6 shows the fitting results of these three functions, from which it can be seen that all these three functions fit well. This paper recommends an exponential function. The exponential function was used to fit Gao Hui’s experimental data [4], and the fitting results are excellent, as shown in Figure 23. It can be seen that the change in aggregate structure did not change the exponential function relationship between total porosity and compressive strength. 

As can be seen from Table 5, the maximum change in the compressive strength of the specimens with different sub-porosities but the same total porosity and aggregate structure is 1.658 MPa, which occurs in the specimen with total porosity of 2%, accounting for 8.4% of its compressive strength. The minimum change is 0.02 MPa, which occurs in the specimen with total porosity of 4%, accounting for 0.12% of its compressive strength. The maximum change in the compressive strength of the specimens with different pore space distribution but the same aggregate structure, total porosity, and sub-porosity is 1.378 MPa, accounting for 6.96% of its compressive strength. The minimum fluctuation amplitude is 0.024 MPa, which occurs between specimens M-3-1 and M-3-2, accounting for 0.13% of its compressive strength.

**Figure 24:**
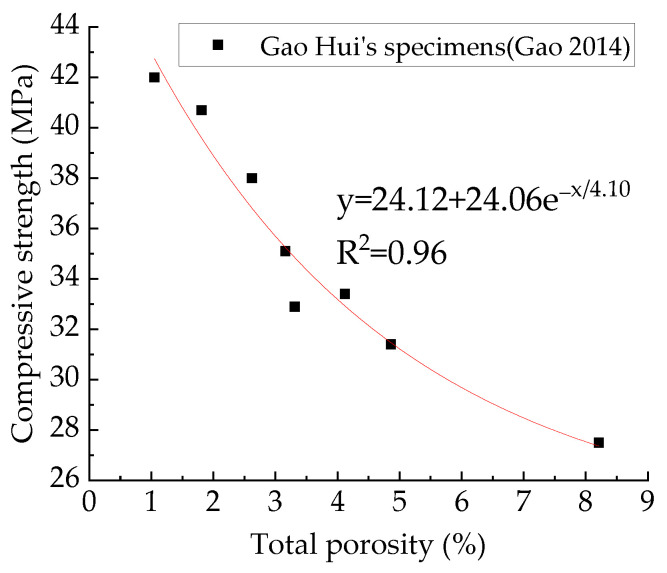
Relationship between compressive strength and total porosity of Gao Hui’s specimens [4].

Figure 24 shows the compressive strength of three types of concrete established in this paper. For samples with the same volume and total porosity, the greater the sub-porosity of macropores, the smaller the number of pores contained in the specimen. Macropores tend to weaken the strength of the specimen, but a decrease in the number of pores tends to strengthen the specimen. Concrete strength is affected by a combination of these two factors, neither of which dominates, and the strength exhibits strong randomness, as shown in Figure 24.

**Figure 25:**
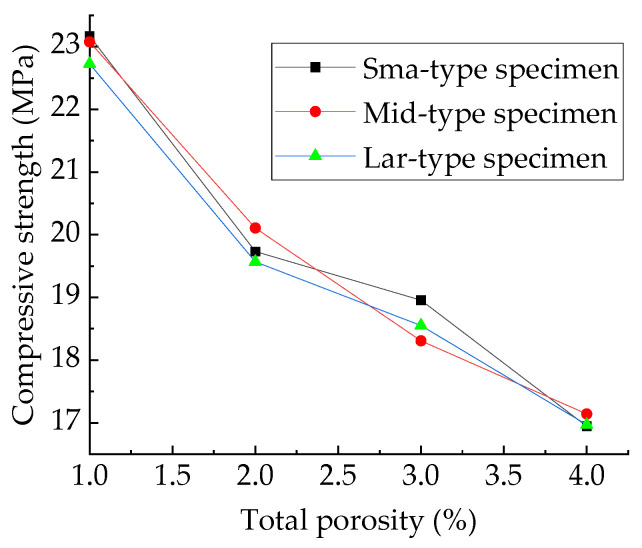
Compressive strength of three types of specimens.

Figure 25 shows the relationship between PSSA and compressive strength. The fitting results of polynomial, exponential, and power functions are shown in Table 7, from which it can be found that all these three functions can fit well. This paper recommends a power function. It can be found from Figure 25 that for the specimens with the same aggregate structure and total porosity, the effect of PSSA on compressive strength does not show regularity. Related studies [80,81,82] concluded that the strength of concrete decreases with increasing PSSA, which is the same as the results of this paper. However, they did not study according to the control variable method, and the conclusions reached stayed on qualitative analysis rather than quantitative analysis. The research on the relationship between PSSA and concrete strength based on the control variable method has not been reported yet.

**Figure 26:**
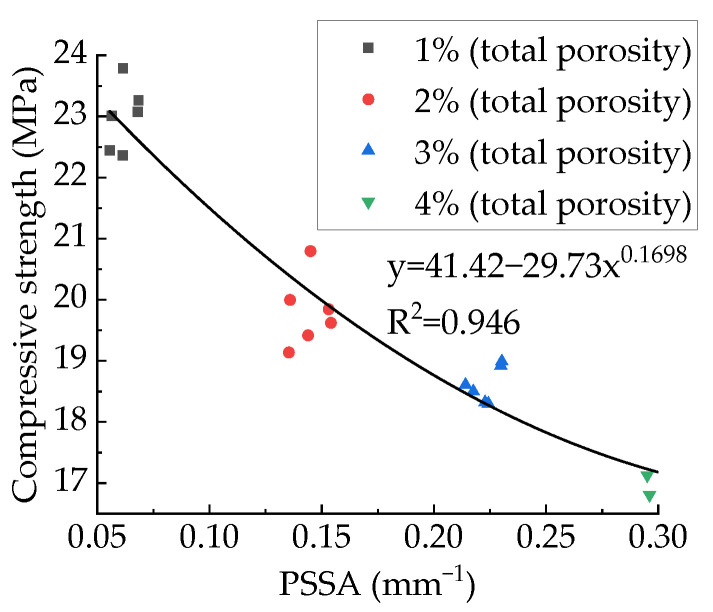
Relationship between PSSA and compressive strength.

Figure 26 shows the relationship between the APR and the compressive strength. It can be found that the APR decreases as the total porosity increases. For specimens with the same volume and total porosity, the higher the macropore content, the larger the APR. With the increase in the APR, the compressive strength tends to increase, but this is due to a decrease in the total porosity. For specimens with the same aggregate structure and total porosity, the compressive strength is largely unaffected by APR, as shown in Figure 26, which is consistent with Du Xiuli’s findings [2].

### 3.3. Effect of Pore Structure on the Compressive Elastic Modulus

**Figure 27:**
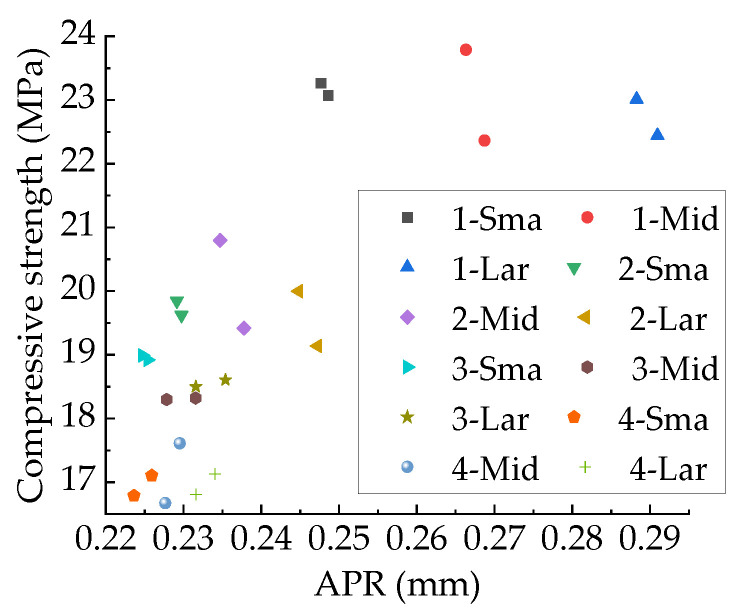
Relationship between APR and compressive strength. The letters in the legend represent concrete types and the numbers represent total porosity.

The experimental study [78] and numerical simulation experimental studies [41,83] found that the exponential and linear functions can well describe the relationship between modulus and total porosity. However, in these experiments, both the total porosity and aggregate structure changed. The relationship between compressive elastic modulus and total porosity for samples with different total porosity but the same aggregate structure is shown in Figure 27. The fitting results of exponential function, linear function and power function are shown in Table 8. It can be found that all these three types of functions can well fit the relationship between the compressive modulus and the total porosity. Considering the simplicity of the functional relationship, this paper recommends a linear function. This is consistent with Xiao’s findings [84] and Zhu’s findings [83]. It can be seen that the linear function relationship between compressive modulus and total porosity is basically unaffected by aggregate distribution.

As can be seen from Table 5, the maximum and minimum fluctuation amplitudes of the compressive modulus of the specimens with different sub-porosities but the same aggregate structure and total porosity are 1809 MPa and 288 MPa, respectively. The maximum occurs in the specimen with total porosity of 1%, accounting for 5% of its compressive elastic modulus. The fluctuation amplitude tends to decrease with the increase in the total porosity. The minimum occurs in the specimen with total porosity of 4%, accounting for 1% of its compressive modulus. The maximum change in the compressive modulus of the specimens with different pore space distribution but the same aggregate structure, total porosity, and sub-porosity is 648 MPa, which occurs between specimens M-1-1 and M-1-2, accounting for 2% of its compressive modulus.

**Figure 28:**
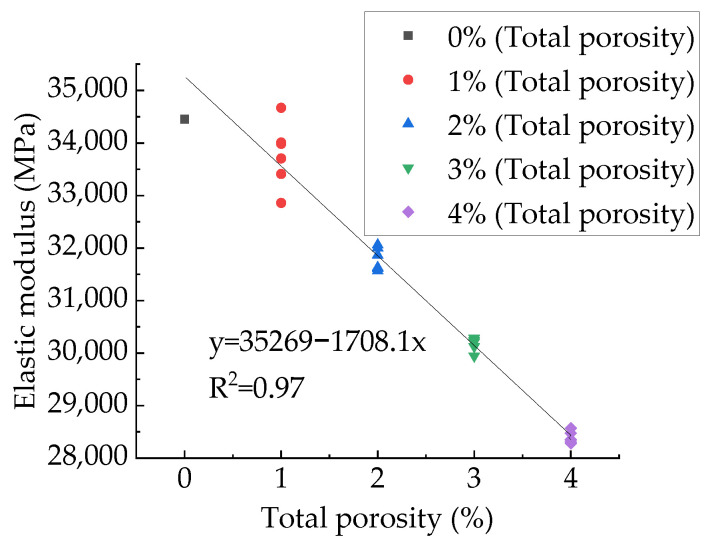
Relationship between compressive modulus and total porosity.

The moduli of the three types of specimens are shown in Figure 28. The greater the sub-porosity of macropores of the samples with the same volume and total porosity, the lower the number of pores. Macropores in the specimen tend to lower the elastic modulus of the specimen, while a reduction in the number of pores tends to increase the elastic modulus. The elastic modulus is affected by a combination of these two factors, neither of which dominates, and the elastic modulus exhibits strong randomness, as shown in Figure 28.

**Figure 29:**
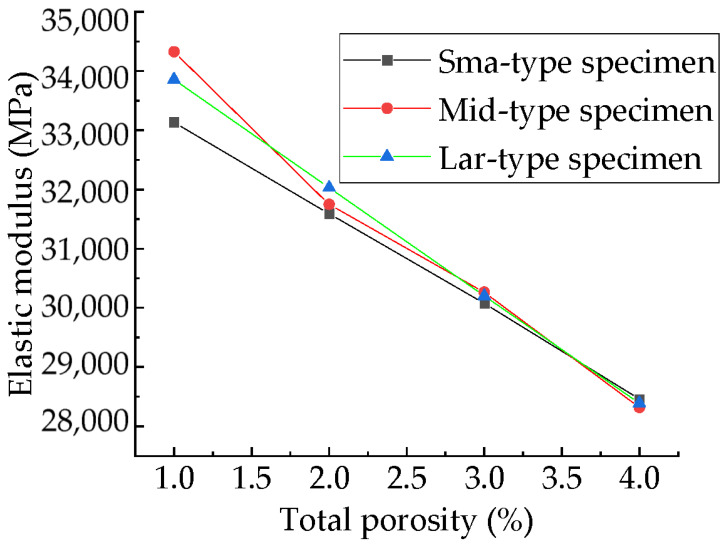
Compressive moduli of three types of specimens.

**Figure 30:**
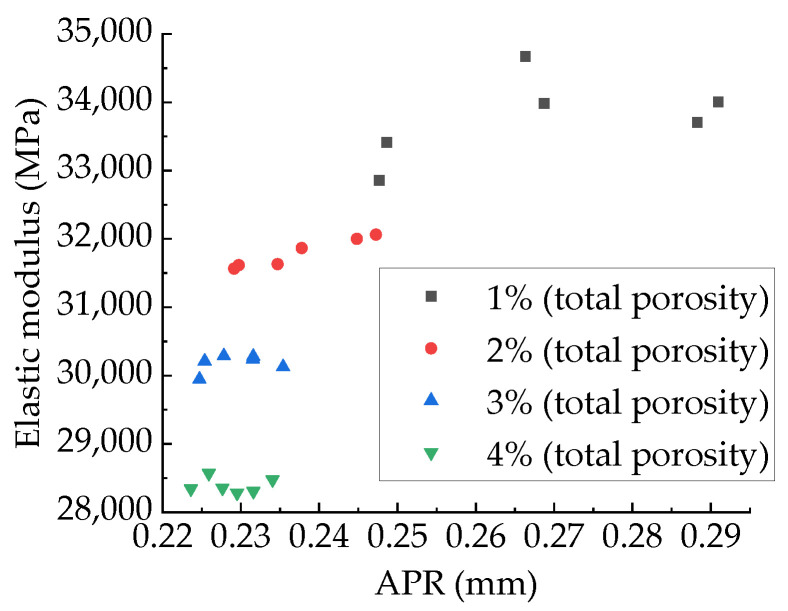
Relationship between APR and compressive modulus.

Figure 29 shows the relationship between APR and modulus, and Figure 30 shows the relationship between modulus and PSSA. It can be found from Figure 29 that for the specimens with the same aggregate structure and total porosity, the modulus is basically unaffected by the APR, which is the same as Du Xiuli’s research results [2]. The linear, exponential, and power functions are used to fit the relationship between PSSA and compressive elastic modulus, and the results are shown in Table 9. It can be found that all these three types of functions can well fit the relationship between PSSA and compressive modulus. Considering the simplicity of the functional relationship, this paper recommends a linear function.

### 3.4. Effect of Pore Structure on Peak Strain of Concrete

**Figure 31:**
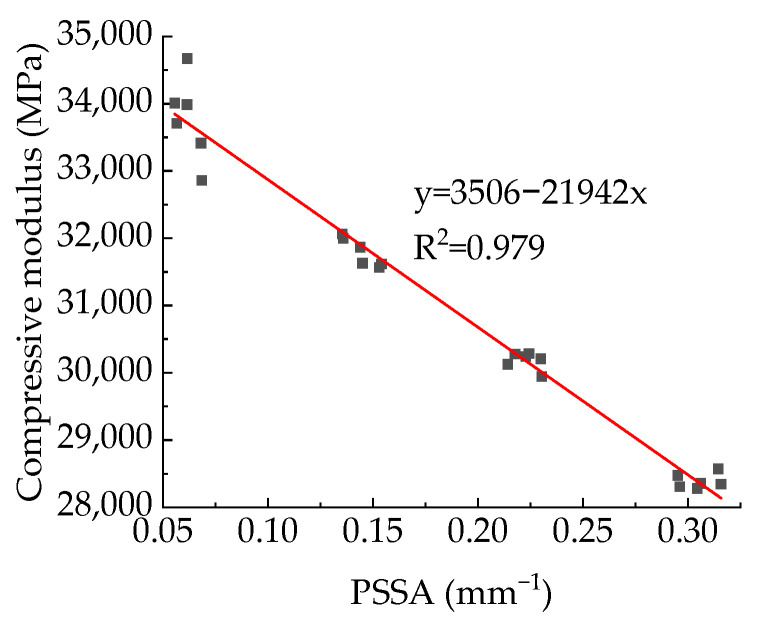
Relationship between compressive modulus and PSSA.

The relationship between peak strain and total porosity are shown in Figure 31. Taking the average value as the calculated value of the mechanical parameters, the peak strains of the specimens with different total porosity are shown in Table 10. It can be seen from Table 10 that when the total porosity changes by 1%, the maximum change in peak strain is 0.163 με, and the minimum change is 0.0047 με. It can be seen from Table 5 that the maximum fluctuation amplitude of peak strain of the specimens with different sub-porosities but the same aggregate structure and total porosity is 0.238 με, which occurs between specimens M-2-2 and L-2-1, accounting for 23.5% of its peak strain. The maximum fluctuation amplitude of peak strain of the specimens with different pore space distribution but the same total porosity and sub-porosity is 0.181 με, which occurs between specimens M-2-1 and M-2-2, accounting for 17.3% of its peak strain. From this, it can be judged that sub-porosity and pore space distribution have stronger effects on peak strain than total porosity.

**Figure 32:**
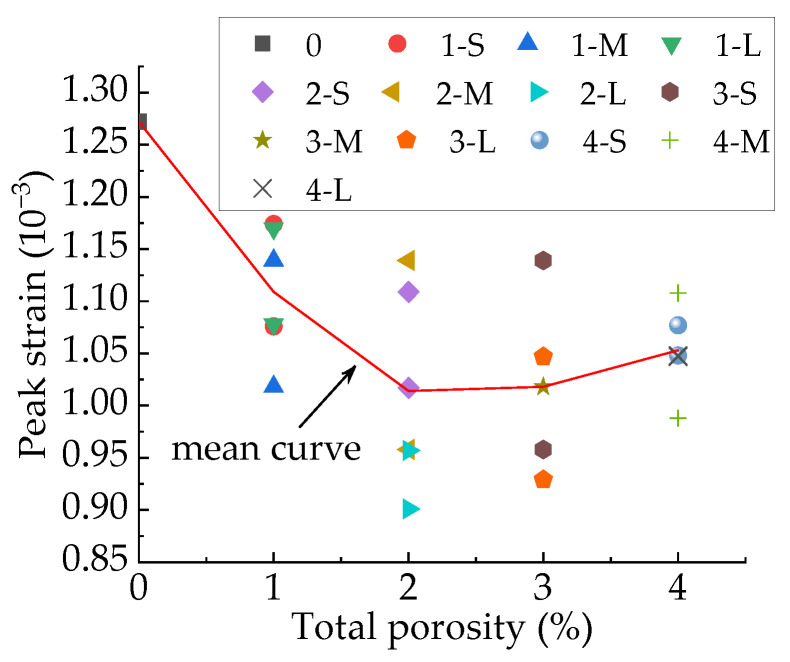
Relationship between peak strain and total porosity.

**Figure 33:**
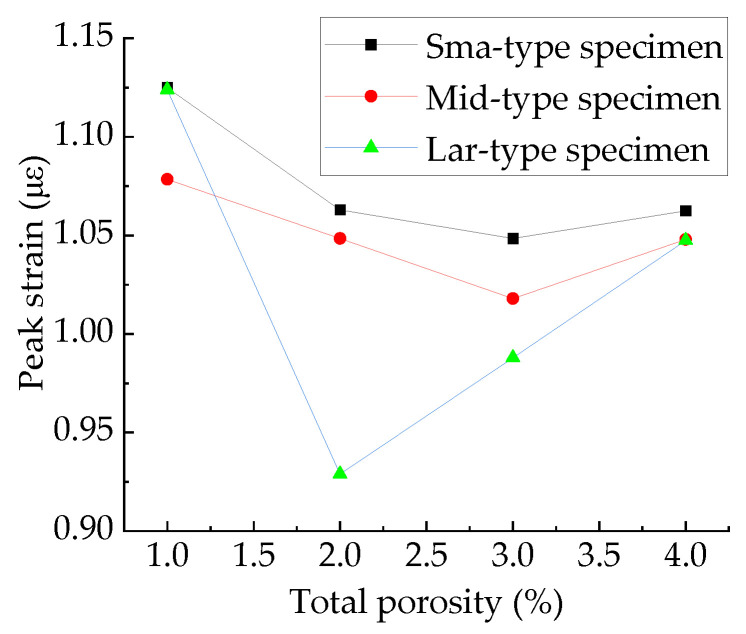
Peak strain of three types of specimens.

**Figure 34:**
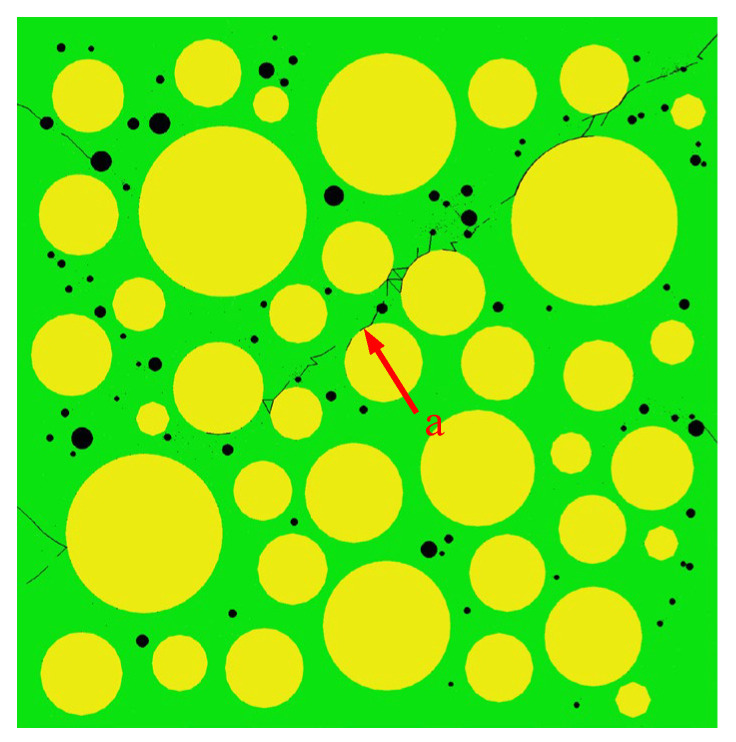
The first macroscopic crack of the L-1-1 specimen.

**Figure 35:**
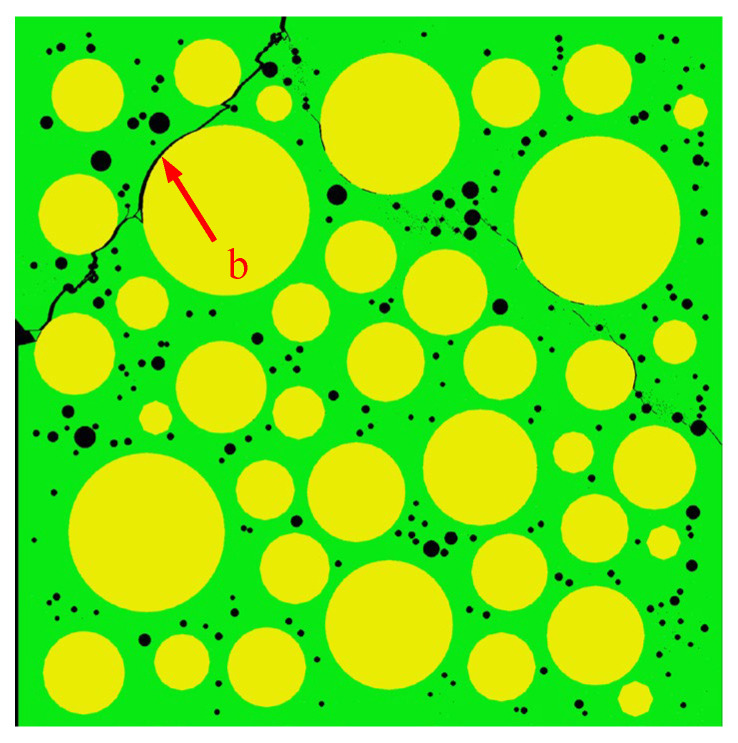
The first macroscopic crack of the L-2-1 specimen.

Figure 32 shows the peak strain of three types of concrete. It can be seen from Figure 32 that the peak strain of the Sma-type specimen is larger than that of the Mid-type specimen. For specimens with the same total porosity, the Sma-type specimens have more small pores, fewer macropores, and higher compactness than the Mid-type specimens. This may be the reason why the Sma-type specimen is more ductile than the Mid-type specimen, i.e., the peak strain is larger. However, the Lar-type specimen does not continue this trend. It can be seen from Figure 32 that the peak strain of the Sma-type specimen and the Mid-type specimen is basically not affected by the total porosity, while the peak strain of the Lar-type specimen fluctuates greatly with the change in the total porosity. The peak strain of the Lar-type specimen with total porosity = 1% is large enough to be comparable to that of the Sma-type specimen, and then decreases sharply as the total porosity becomes 2%. It can be seen from Table 5 that the peak strain of the specimen L-1-1 is 1.170 × 10^−3^, which is close to the maximum value of the peak strain of all specimens. This is why the peak strain of the Lar-type specimen with total porosity = 1% is comparable to that of the Sma-type specimen. The peak strain of the specimen L-2-1 is 0.901 × 10^−3^, which is the minimum value of the peak strain of all specimens. This is why the Lar-type specimen with total porosity = 2% drops sharply. By studying the visual cracking process of the specimen L-1-1 and the specimen L-2-1, it is found that the peak strain is closely related to the morphology of the first macroscopic crack of the specimen. After the specimen reaches peak strain, the first macroscopic crack germinates in the most damaged areas in the cement mortar. Therefore, the morphology of the first macroscopic crack can reflect the damage of the specimen when it is under peak stress to a certain extent. If the first macroscopic crack passes through the middle of the specimen and has a long length, the specimen often exhibits strong ductility and has a large peak strain, such as the L-1-1 specimen. We call this morphology of the first macroscopic crack Mode 1. Figure 33 shows the first macroscopic crack of the specimen L-1-1, marked as a. If the first macroscopic crack appears at the end of the specimen and penetrates the specimen with a short length, the specimen is prone to brittle failure and has a small peak strain, such as the specimen L-2-1. We call this morphology of the first macroscopic crack Mode 2. Figure 34 shows the first macroscopic crack of the specimen L-2-1, marked as b. Similar patterns were found in the peak strain and failure of other specimens such as Specimen S-3-1 and Specimen S-3-2. Figure 35 show the first macroscopic cracks of Specimen S-3-1 and Specimen S-3-2, marked as c and d, respectively. Specimen S-3-2 and Specimen S-3-1 have the same aggregate structure, total porosity, and sub-porosity but different pore spatial distributions. Specimen S-3-1 belongs to Mode 1 with a large peak strain of 1.139 × 10^−3^. Specimen S-3-2 belongs to Mode 2 with a small peak strain of 0.958 × 10^−3^, which is 0.181 × 10^−3^ less than that of the S-3-1 specimen. The peak strain and the morphology of the first macroscopic crack are affected by many factors, such as porosity, pore spatial distribution, and aggregate structure. As can been seen from the large difference between the peak strains of specimen L-3-1 and specimen L-3-2, the influence of pore spatial distribution on the peak strain cannot be ignored.

**Figure 36:**
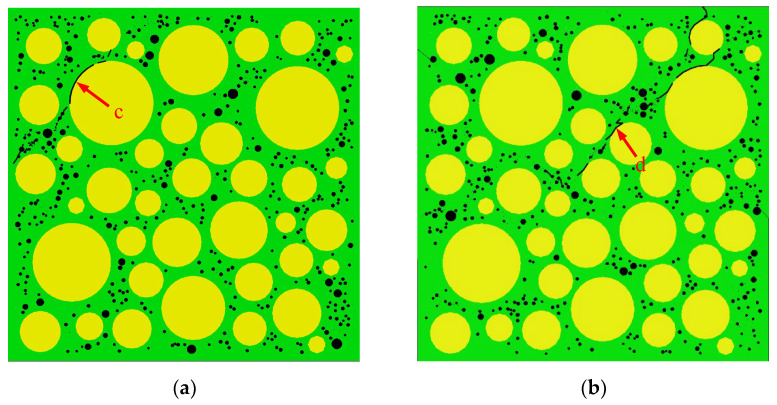
The first macroscopic cracks of Specimen S-3-1 and Specimen S-3-2. (**a**) S-3-2; (**b**) S-3-1.

**Figure 37:**
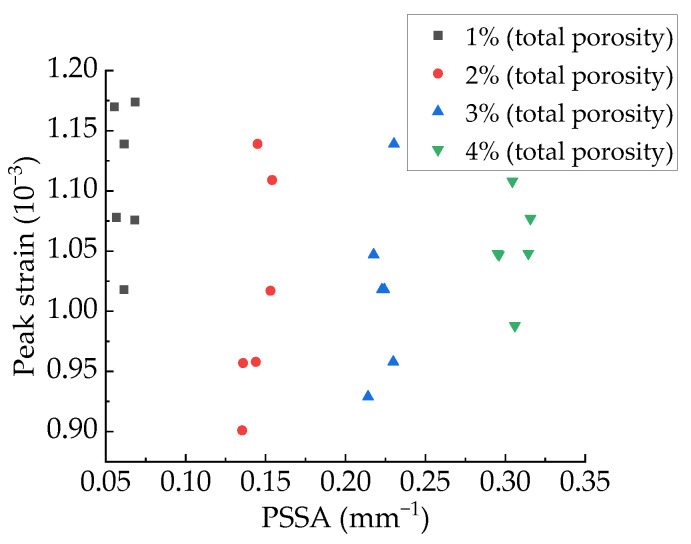
Relationship between peak strain and PSSA.

**Figure 38:**
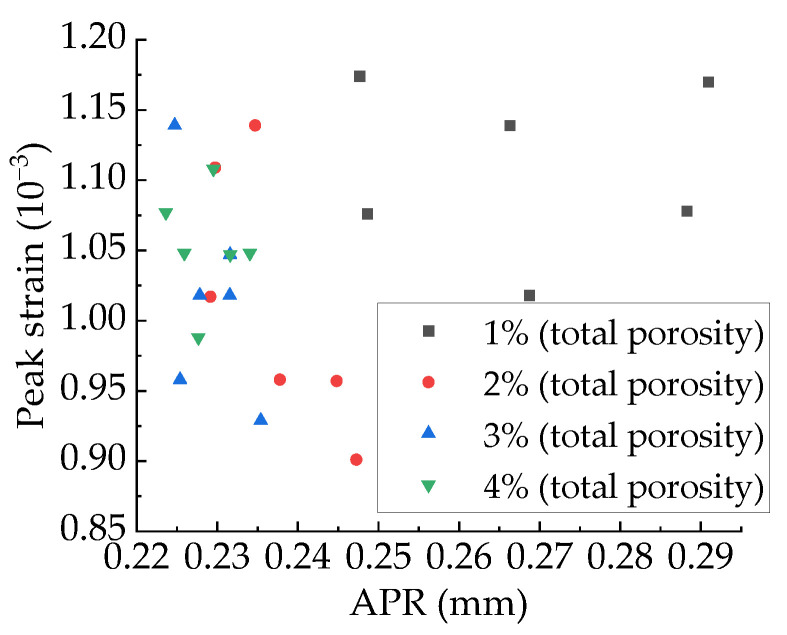
Relationship between APR and peak strain.

The relationship between peak strain and PSSA are shown in Figure 36, and the relationship between APR and peak strain are shown in Figure 37. It can be found that the PSSA and APR have no regular effect on peak strain (Figure 38).

### 3.5. Grey Relational Analysis (GRA) of Concrete Mechanical Behaviors

When the number of the sub-sample is too small to fully present the maternal information, the method of mathematical–statistical analysis lacks accuracy, and it is prone to inconsistency between the quantitative results and the qualitative analysis. In this case, the grey system analysis method should be used. GRA is a method to compare the degree of correlation among factors according to the degree of similarity or degree of dissimilarity in the development trend of factors, which is proposed by Deng Julong [85]. It is usually used to analyze the relationship among objects with ambiguous internal connotation, incomplete operating mechanism, and sparse behavior data. GRA is used to explore the effect of pore structure on the concrete mechanical behavior under uniaxial compressive loading, and the total porosity, sub-porosity of each pore gradation segment, APR, and PSSA are taken as the comparison columns. Compressive strength, peak strain, and modulus are used as the reference columns, respectively. The specific calculation process is shown in Reference [85] and the calculated relational grades are shown in Table 11.

It can be found from Table 11 that the descending order of the relational grades between pore structure parameters and compressive strength is: total porosity > T [k_1_,k_2_] > T [k_2_,k3] > T [k_3_,k_4_] > T [k_4_,k_5_] > APR > PSSA. For pores with a diameter of 0.3~1.6 mm, the larger the pore size, the smaller the effect of its content on the compressive strength. Compressive strength is most affected by total porosity. The pore structure parameters studied in this paper have the same sequential effect on the elastic modulus and compressive strength of concrete. The descending order of influence of pore structure parameters on peak strain is: T [k_2_,k_3_] > T [k_1_,k_2_] > T [k_3_,k_4_] > T [k_4_,k_5_] > APR > PSSA > total porosity. Among the pore structure parameters studied, total porosity is the least important factor affecting peak strain, but the most important factor affecting compressive strength and modulus. Peak strain is most affected by sub-porosity of pores with radii in the range of 0.25~0.4 mm. Among the pore structure parameters considered in this paper, PSSA has the least influence on the compressive strength and modulus. However, from the analysis in Section 3.2 and Section 3.3, it can be seen that there are obvious functional relationships between modulus and PSSA, and between compressive strength and PSSA, which is of great significance for quantitative analysis of the effect of pore structure on elastic modulus and strength. Furthermore, the influence of total porosity on peak strain is smaller than that of PSSA. Therefore, it is necessary to use PSSA as a parameter to describe the pore structure and explore the effect of pore structure on the mechanical behavior of concrete.

## 4. Conclusions

A novel CRAMM is proposed to model the pore structure by parameters such as pore gradation, total porosity, pore size, and sub-porosity of each pore gradation. To study the effect of pore structure on the mechanical behavior of concrete, 25 mesoscopic concrete specimens with different pore structures but the same aggregate structure are established and subjected to uniaxial compression tests. The effects of total porosity, sub-porosity of each pore gradation, PSSA, and APR on the concrete cracking process, modulus, peak strain, and compressive strength are investigated.

(1) Under uniaxial compressive loading, the cracking process of specimens with pores and specimens without pores is very similar. The damage first germinates at the periphery of the sample and then expands toward the center. The damage of the ITZ spreads to the center of the specimen in the relatively stable stage of the micro-cracks, which is faster than that of cement mortar. The damage of cement mortar spreads toward the center of the sample in the stable development stage of the micro-cracks.
(2) The presence of the pore structure does not accelerate this expansion process, nor does it change the phenomenon that the most damaged area in the concrete before peak stress is the ITZ. However, the pore structure makes the germination and propagation of the damage in cement mortar show obvious locality. The initiation and propagation of macroscopic cracks are greatly affected by the pore structure.
(3) The sudden drops in the descending section of the stress–strain curve are often accompanied by the generation and expansion of macroscopic cracks.
(4) The quadratic polynomial, exponential, and power functions can well fit the relationship between total porosity and compressive strength and the relationship between PSSA and compressive strength.
(5) The linear, exponential, and power functions can well characterize the relationship between compressive modulus and total porosity and the relationship between PSSA and compressive modulus.
(6) For the concrete specimens with the same aggregate structure and total porosity, the modulus and compressive strength show randomness with the increase in the sub-porosity of macropores, and the APR has little effect on compressive strength and modulus.
(7) The influence of pore space distribution and sub-porosity on peak strain is greater than that of total porosity on peak strain. The effects of PSSA and APR on peak strain do not show obvious regularity.
(8) According to the GRA, the pore structure parameters considered in this paper have the same order of influence on the modulus and compressive strength of concrete, which is different from the order of influence on the peak strain. Total porosity has the least effect on peak strain but the largest effect on compressive strength and modulus. For pores with a diameter of 0.3~1.6 mm, the larger the pore size, the smaller the effect of its content on the modulus and compressive strength. The peak strain is most affected by the sub-porosity of pores with radii in the range of 0.25~0.4 mm.

## Figures and Tables

**Table 1:** Aggregate size distribution.

Sieve Size (mm)	Total Percentage Passing (%)
19.00	100
12.70	97
9.50	61
4.75	10
2.36	1.4

**Table 2:** CRAMs and pore structure parameters.

Sample	T [k_1_,k_2_] (%)	T [k_2_,k_3_] (%)	T [k_3_,k_4_] (%)	T [k_4_,k_5_] (%)	Total Porosity (%)	APR (mm)	PSSA (mm^−1^)
0–1 (reference sample)	0	0	0	0	0	0	0
S-1-1	0.4	0.3	0.14	0.15	1	0.247672	0.0684
S-1-2	0.4	0.3	0.14	0.15	1	0.248598	0.0681
M-1-1	0.305	0.3	0.2	0.195	1	0.266321	0.0615
M-1-2	0.305	0.3	0.2	0.195	1	0.268718	0.0614
L-1-1	0.21	0.3	0.25	0.24	1	0.290971	0.0555
L-1-2	0.21	0.3	0.25	0.24	1	0.288282	0.0565
S-2-1	1.1	0.5	0.28	0.12	2	0.229737	0.1541
S-2-2	1.1	0.5	0.28	0.12	2	0.229149	0.1531
M-2-1	0.965	0.49	0.34	0.205	2	0.237766	0.1439
M-2-2	0.965	0.49	0.34	0.205	2	0.234691	0.1450
L-2-1	0.83	0.48	0.4	0.29	2	0.247249	0.1354
L-2-2	0.83	0.48	0.4	0.29	2	0.244804	0.1359
S-3-1	1.75	0.52	0.42	0.31	3	0.224720	0.2303
S-3-2	1.75	0.52	0.42	0.31	3	0.225388	0.2299
M-3-1	1.655	0.485	0.505	0.355	3	0.227832	0.2243
M-3-2	1.655	0.485	0.505	0.355	3	0.231550	0.2228
L-3-1	1.56	0.45	0.59	0.4	3	0.235400	0.2141
L-3-2	1.56	0.45	0.59	0.4	3	0.231589	0.2176
S-4-1	2.28	0.99	0.49	0.24	4	0.223631	0.3157
S-4-2	2.28	0.99	0.49	0.24	4	0.225920	0.3144
4-1-M	2.135	0.97	0.57	0.325	4	0.227655	0.3060
4-2-M	2.135	0.97	0.57	0.325	4	0.229511	0.3044
L-4-1	1.99	0.95	0.65	0.41	4	0.231597	0.2961
4-2-L	1.99	0.95	0.65	0.41	4	0.234038	0.2951

Note: T [k_1_,k_2_]: the sub-porosity of pores in gradation segment [k_i_,k_i+1_], I = 1, 2, 3, 4, [k_1_,k_2_] = [0.15,0.25], [k_2_,k_3_] = [0.25,0.4], [k_3_,k_4_] = [0.4,0.6], [k_4_,k_5_] = [0.6,0.8]; S: Sma-type specimen; L: Lar-type specimen; M: Mid-type specimen.

**Table 3:** Mechanical parameters of the CRAMs.

	Solid Elements		Cohesive Elements	
	Aggregate	Cement Mortar	ITZ	Cement Mortar
Density (10^−9^ t/mm^3^)	2.5	2.2	2.2	2.2
Elastic modulus (GPa)	70	25	/	/
Poisson’s ratio	0.2	0.2	/	/
Elastic stiffness (MPa/mm)	/	/	1,100,000	1,100,000
Tensile strength (MPa)	/	/	3.9	11.7
Fracture energy (N/mm)	/	/	0.039	0.117

**Table 4:** The comparison results.

	Modulus (GPa)	Error (%)	Strength (MPa)	Error (%)
Experimental specimens [69]	34.7		31	
Numerical specimens [34]	32.4	6.6	31.4	1.3
Specimen in this paper	34.6	0.3	31.6	1.9

**Table 5:** Mechanical properties of 25 CRAMs.

Sample	Modulus (MPa)	Strength (MPa)	Peak Strain (με)
0-1	34,451	26.106	1.272
S-1-1	32,860	23.263	1.174
S-1-2	33,412	23.075	1.076
M-1-1	34,669	23.788	1.139
M-1-2	33,985	22.363	1.018
L-1-1	34,009	22.441	1.170
L-1-2	33,707	23.010	1.078
S-2-1	31,616	19.622	1.109
S-2-2	31,564	19.845	1.017
M-2-1	31,866	19.418	0.958
M-2-2	31,629	20.796	1.139
L-2-1	32,062	19.138	0.901
L-2-2	32,000	19.998	0.957
S-3-1	29,944	18.991	1.139
S-3-2	30,209	18.921	0.958
M-3-1	30,287	18.297	1.018
M-3-2	30,241	18.321	1.018
L-3-1	30,126	18.606	0.929
L-3-2	30,279	18.499	1.047
S-4-1	28,343	16.788	1.077
S-4-2	28,570	17.104	1.048
4-1-M	28,355	16.673	0.988
4-2-M	28,282	17.611	1.108
L-4-1	28,308	16.808	1.047
4-2-L	28,476	17.129	1.048

**Table 6:** Fitting results of the relationship between compressive strength and total porosity.

Function Type	Fit Function	Correlation Coefficient (R^2^)
Polynomial function	y = 0.375x^2^ − 3.776x + 26.227	0.9635
Exponential function	y = 14.04 + 12.39e^−x/2.846^	0.964
Power function	y = 831.54 − 808.61x^0.0052^	0.959

**Table 7:** Fitting results of the relationship between PSSA and compressive strength.

Function Type	Fit Function	Correlation Coefficient (R^2^)
Polynomial function	y = 25.38 − 44.56x^2^ + 57.39x	0.942
Exponential function	y = 15.08 + 10.944e^−x/0.1835^	0.945
Power function	y = 41.42 − 29.73x^0.1698^	0.946

**Table 8:** Fitting results of the relationship between compressive modulus and total porosity.

Function Type	Fit Function	Correlation Coefficient (R^2^)
Linear function	y = 35,269 − 1708.1x	0.970
Exponential function	y = 54,731 − 19,701e^x/13.706^	0.972
Power function	y = 36,091 − 2336x^0.858^	0.976

**Table 9:** Fitting results of the relationship between PSSA and compressive modulus.

Function Type	Fit Function	Correlation Coefficient (R^2^)
Linear function	y = 3506 − 21,942x	0.979
Exponential function	y = 8259 + 27,089e^−x/1.036^	0.980
Power function	y = 35,720 − 19,509x^0.830^	0.980

**Table 10:** The peak strains of the specimens with different total porosity.

Total Porosity	0%	1%	2%	3%	4%
Peak strain	1.2720	1.1092	1.0135	1.0182	1.0527

**Table 11:** Relational grades.

Project	Total Porosity	T [k_1_,k_2_]	T [k_2_,k_3_]	T [k_3_,k_4_]	T [k_4_,k_5_]	APR	PSSA
Elastic modulus	0.999873	0.999847	0.999832	0.999829	0.999826	0.999825	0.999824
Strength	0.8501	0.81425	0.79458	0.79041	0.7869	0.78596	0.7849
Peak strain	0.72243	0.81349	0.8259	0.7939	0.76944	0.7661	0.75169

## Data Availability

Not applicable.
